# ORIUM: Optimized RDC-based Iterative and Unified Model-free analysis

**DOI:** 10.1007/s10858-013-9775-1

**Published:** 2013-09-08

**Authors:** T. Michael Sabo, Colin A. Smith, David Ban, Adam Mazur, Donghan Lee, Christian Griesinger

**Affiliations:** 1 Department for NMR-based Structural Biology, Max-Planck Institute for Biophysical Chemistry, Am Fassberg 11, 37077 Göttingen, Germany; 2 Department of Theoretical and Computational Biophysics, Max-Planck Institute for Biophysical Chemistry, Am Fassberg 11, 37077 Göttingen, Germany

**Keywords:** RDCs, Dynamics, Ubiquitin, GB3

## Abstract

Residual dipolar couplings (RDCs) are NMR parameters that provide both structural and dynamic information concerning inter-nuclear vectors, such as N–H^N^ and Cα–Hα bonds within the protein backbone. Two approaches for extracting this information from RDCs are the model free analysis (MFA) (Meiler et al. in J Am Chem Soc 123:6098–6107, 2001; Peti et al. in J Am Chem Soc 124:5822–5833, 2002) and the direct interpretation of dipolar couplings (DIDCs) (Tolman in J Am Chem Soc 124:12020–12030, 2002). Both methods have been incorporated into iterative schemes, namely the self-consistent RDC based MFA (SCRM) (Lakomek et al. in J Biomol NMR 41:139–155, 2008) and iterative DIDC (Yao et al. in J Phys Chem B 112:6045–6056, 2008), with the goal of removing the influence of structural noise in the MFA and DIDC formulations. Here, we report a new iterative procedure entitled Optimized RDC-based Iterative and Unified Model-free analysis (ORIUM). ORIUM unifies theoretical concepts developed in the MFA, SCRM, and DIDC methods to construct a computationally less demanding approach to determine these structural and dynamic parameters. In all schemes, dynamic averaging reduces the actual magnitude of the alignment tensors complicating the determination of the absolute values for the generalized order parameters. To readdress this scaling issue that has been previously investigated (Lakomek et al. in J Biomol NMR 41:139–155, 2008; Salmon et al. in Angew Chem Int Edit 48:4154–4157, 2009), a new method is presented using only RDC data to establish a lower bound on protein motion, bypassing the requirement of Lipari–Szabo order parameters. ORIUM and the new scaling procedure are applied to the proteins ubiquitin and the third immunoglobulin domain of protein G (GB3). Our results indicate good agreement with the SCRM and iterative DIDC approaches and signify the general applicability of ORIUM and the proposed scaling for the extraction of inter-nuclear vector structural and dynamic content.

**Electronic supplementary material:**

The online version of this article (doi:10.1007/s10858-013-9775-1) contains supplementary material, which is available to authorized users.

## Introduction

Protein structure and dynamics are routinely investigated with NMR spectroscopy at atomic resolution. An essential NMR parameter that provides both structural and dynamic information is the residual dipolar coupling (RDC) between two nuclear magnetic moments, for example a N–H^N^ or Cα–Hα vector within the polypeptide backbone of a protein (Tjandra and Bax 1997; Tolman et al. 1997). An important application of RDCs as a probe for protein dynamics has been shown recently where RDCs measured in 36 different alignment media demonstrated that the ground state conformational ensemble of ubiquitin covers the conformational space captured in crystal structures of ubiquitin complexes (Lange et al. 2008; Lakomek et al. 2008). These findings provide strong support for conformational selection as a means for molecular recognition. Therefore, extracting the dynamical content from RDCs has implications for understanding the mechanisms of molecular recognition and protein function.

The significance of the RDC’s dynamical content is highlighted when considering other approaches for studying protein dynamics. Measurements of NMR spin-relaxation provide information concerning amplitudes of inter-nuclear vector motions occurring on time-scales faster than the rotational correlation time (τ_c_) of the protein (picosecond to nanosecond) (Kay et al. 1989), which are parameterized by the Lipari–Szabo order parameter (*S*_*LS*_^2^) (Lipari and Szabo 1982a, b). Relaxation dispersion methods probe the kinetics of conformational exchange that modulates the isotropic chemical shift and contributes to the effective transverse relaxation rate (Palmer 2004). To date, relaxation dispersion techniques have been limited to time-scales slower than approximately 25 μs (Ban et al. 2011, 2012). By contrast, RDCs provide vital insight into the amplitude and direction of internal vector motions on time-scales covering the previously inaccessible time window spanning τ_c_ to ~25 μs (referred to as the supra-τ_c_ window).

Residual dipolar couplings (RDCs) arise from placing a protein in an anisotropic medium, such as filamentous phages or lipid bilayers, or paramagnetic tagging, leading to partial alignment of the protein with respect to the external magnetic field. In the anisotropic media, all possible orientations for an inter-nuclear vector are populated with unequal probability, resulting in the dipolar couplings no longer averaging to zero. The magnitude of the measured RDC is given by the time-averaged angle between the inter-nuclear vector and the magnetic field (Tolman et al. 1997).

**Fig. 1:**
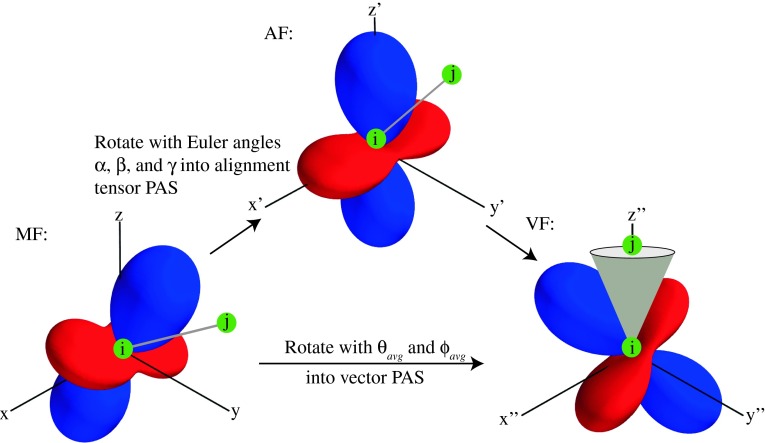
Illustration of the molecular frame (MF), the alignment frame (AF), and the vector frame (VF) and the relationship between the different frames of reference. Conceptually, the SCRM procedure moves from the MF to the AF to the VF, while ORIUM goes directly from the MF to the VF. The alignment tensor is depicted with the positive lobes in *blue* and the negative lobes in *red*

**Fig. 2:**
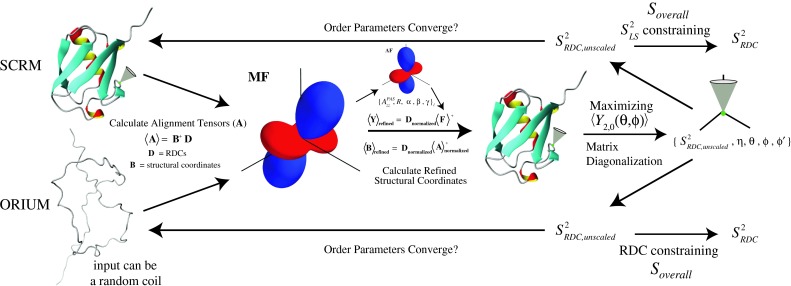
Schematic diagram of both ORIUM and SCRM protocols (Lakomek et al. 2008). Both procedures begin with a starting structure as input and use experimental RDCs to calculate the alignment tensors in the molecular frame (MF). In SCRM, the principal axis system of each alignment tensor is determined in order to extract $\left\{ {A_{zz}^{PAS} ,R,\alpha ,\beta ,\gamma } \right\}_{l}$ which are used to construct the **F** matrix and scale the RDCs. With ORIUM, only $A_{zz,l}^{PAS}$ is needed to scale the RDCs. The next step is to calculate refined structural coordinates. SCRM requires the five alignment tensor parameters $\left\{ {A_{zz}^{PAS} ,R,\alpha ,\beta ,\gamma } \right\}_{l}$ to construct the **F** matrix for the determination of $\left\langle {\mathbf{Y}} \right\rangle_{{{\mathbf{refined}}}}$. ORIUM determines $\left\langle {\mathbf{B}} \right\rangle_{{{\mathbf{refined}}}}$ directly from $\left\langle {\mathbf{A}} \right\rangle_{{{\mathbf{normalized}}}}$. In order to calculate $\left\{ {S_{RDC}^{2} ,\eta ,\theta_{{}}^{MF} ,\phi_{{}}^{MF } ,\phi^{'} } \right\}_{k}$, SCRM maximizes $\left\langle {Y_{2,0} (\theta_{k}^{VF} ,\phi_{k}^{VF} )} \right\rangle$ while ORIUM utilizes Saupe matrix diagonalization (Meirovitch et al. 2012). The angles $(\theta_{k}^{MF} ,\phi_{k}^{MF} )$ are then used as input to restart the SCRM and ORIUM protocols until $S_{RDC, unscaled}^{2}$ converges. Once this criterion is fulfilled, the $S_{overall}$ parameter is determined, which scales the order parameters

Since the potential to extract dynamics from RDCs was first recognized, two schemes for extracting the dynamical content from these NMR parameters in the form of a generalized order parameter $(S_{RDC}^{2} )$ have been proposed. In the model free analysis (MFA), five independent alignment media are necessary to calculate the five independent elements of the inter-nuclear vector tensor (Meiler et al. 2001; Peti et al. 2002). Figure 1 illustrates the three frames of reference used in the analysis of RDCs, the molecular frame (MF), the alignment frame (AF), and the vector frame (VF). Knowledge of the protein structure is necessary to determine the alignment tensors. With the alignment tensor information, the averages over the second rank spherical harmonics describing the mean orientations of the vectors, contained within the inter-nuclear vector tensor $\left( {\left\langle {Y_{ 2,m} (\theta , \phi )} \right\rangle } \right)$, see also Fig. 2), provide the desired structural and dynamic content. An alternative approach, the direct interpretation of dipolar couplings (DIDCs), was developed to bypass the need for structural input in the calculation of the inter-nuclear vector’s structural and dynamic content (Tolman 2002). Five independent alignment media are also necessary for the DIDC method. A single matrix equation is employed to represent the RDC data obtained in multiple alignment media. The inter-nuclear vector tensors are optimized simultaneously and variation in $S_{RDC}^{2}$ is minimized.

Both the MFA and DIDC methods have been incorporated into iterative schemes with the goal of improving the accuracy of the alignment tensor calculation by reducing the effects of the structural noise, termed the self-consistent RDC based MFA (SCRM) and iterative DIDC (Lakomek et al. 2008; Yao et al. 2008). The iterative schemes achieve this by using the refined dynamically averaged coordinates as input for additional runs of either MFA or DIDC, however each approach, as implemented, relies on computationally expensive procedures. In the iterative DIDC method, a grid search is performed which minimizes the difference between the vector’s coordinates and a pool of possible solutions built from an exhaustive list of (*θ*, *ϕ*) combinations to find dynamically averaged coordinates (Yao et al. 2008). As for SCRM, the dynamic average orientation of each vector is calculated by performing a coordinate transformation with maximization of $\left\langle {Y_{ 2,0} (\theta , \phi )} \right\rangle$ (Lakomek et al. 2008). Recently, it has been shown that this transformation can be replaced with the diagonalization of the local ordering Saupe matrix (Meirovitch et al. 2012), which is computationally less demanding.

Here, we describe a new iterative procedure for extracting structural and dynamic information from RDCs entitled Optimized RDC-based Iterative and Unified Model-free analysis (ORIUM). ORIUM unifies the theoretical concepts developed in the MFA, SCRM, and DIDC methods. In addition, a new method is presented to establish a lower bound on protein motion using RDC data alone without requiring a separate determination of $S_{LS}^{2}$. While this has been achieved previously (Salmon et al. 2009) based on several sets of RDCs assuming Gaussian fluctuations, the method introduced here works on a single set of RDCs and does not require a motional model. The applicability of ORIUM and the new scaling procedure are tested with the model proteins ubiquitin and the third immunoglobulin domain of protein G (GB3).

## Theory

Optimized RDC-based Iterative and Unified Model-free analysis (ORIUM) consists of three principal stages for the extraction of RDC order parameters $\left( {S_{RDC}^{2} } \right)$ from data measured in multiple alignment media (see Fig. 2 for schematic diagram). First, the matrix formalism introduced by Tolman in the DIDC approach is utilized to calculate refined structural coordinates from the alignment tensors (Tolman 2002). From here, each refined vector is put into a local axis system in order to determine the vector specific structural and dynamic information (Meirovitch et al. 2012; Meiler et al. 2001; Peti et al. 2002). Finally, the resulting Euler angles are used as structural input to restart the calculation in an iterative fashion, similar to SCRM (Lakomek et al. 2008). ORIUM continues until the variation in $\left( {S_{RDC}^{2} } \right)$ for the entire dataset falls below a certain threshold.

### Alignment tensor calculation

For two nuclear spins, the observed resonance splitting (Hz) resulting from the partial alignment of a protein emanate from the secular part of the magnetic dipole interaction

1 $$ D_{k}^{exp} = D_{ij}^{max} \left\langle {{{\left( {3  \cos^{2} \theta_{k} - 1} \right)} \mathord{\left/ {\vphantom {{\left( {3  \cos^{2} \theta_{k} - 1} \right)} 2}} \right. \kern-0pt} 2}} \right\rangle $$

1a $$ D_{ij}^{max} = - \frac{{\mu_{0} \gamma_{i} \gamma_{j} \hbar }}{{4\pi^{2} r_{ij}^{3} }} $$

where $\mu_{0}$ is the permeability of vacuum, $\gamma_{X}$ is the gyromagnetic ratio of spin *X*, *ℏ* is Planck’s constant, *r*_*ij*_ is the distance between nuclei *i* and *j* (assumed to be fixed at 1.02 Å for the N–H^N^ and 1.095 Å for the Cα–Hα vectors), and *θ*_*k*_ is the angle between the inter-nuclear vector formed by nuclear spin pair *k* and the magnetic field (*B*_0_). The angular brackets denote ensemble averaging. As Eq. (1) explicitly illustrates, the magnitude of $D_{k}^{exp}$ depends on $\left\langle {(3\cos^{2} \theta_{k} - 1)/2} \right\rangle$. By definition, the term cos *θ*_*k*_ is the scalar product between an inter-nuclear vector and the vector parallel to *B*_0_.

When considering a rigid molecule, the coordinates of an inter-nuclear vector can be described within an arbitrary reference frame, termed the molecular frame (MF), and defined by three angles, *β*_*x*_, *β*_*y*_, and *β*_*z*_, between the vector and the respective MF axes. In a similar fashion, the vector parallel to *B*_0_ can be expressed by three angles representing the instantaneous orientation of *B*_0_ relative to the MF axes, *α*_*x*_, *α*_*y*_, and *α*_*z*_. Within the MF, $D_{k}^{exp}$ can be recast as

2 $$ D_{k}^{exp} = D_{ij}^{max} \left\langle {B \cdot A} \right\rangle $$

where $\left\langle {B \cdot A} \right\rangle$ is the scalar product of two vectors representing the inter-nuclear orientations (*B*) and the *B*_0_ orientations (*A*). Here, *A* is the alignment tensor and *B* is the inter-nuclear vector tensor. Both *A* and *B* contain five independent terms and are related to a 3 × 3 second rank Cartesian order tensor as follows (Saupe 1964, 1968; Snyder 1965)

3 $$ A = \left[ {a_{zz} , \frac{1}{\sqrt 3 }\left( {a_{xx} - a_{yy} } \right),\;\frac{2}{\sqrt 3 }a_{xz} ,\;\frac{2}{\sqrt 3 }a_{yz} ,\;\frac{2}{\sqrt 3 }a_{xy} } \right]_{l} $$

where the orientation of *B*_0_ in the MF is given by

3a $$ a_{mn} = \left\langle {\frac{1}{2}\left( {3\cos \alpha_{m} \cos \alpha_{n} - \delta_{mn} } \right)} \right\rangle_{l} $$

and

4 $$ B = \left[ {b_{zz}^{k}, \frac{1}{\sqrt 3 }\left( {b_{xx}^{k} - b_{yy}^{k} } \right), \frac{2}{\sqrt 3 }b_{xz}^{k}, \frac{2}{\sqrt 3 }b_{yz}^{k}, \frac{2}{\sqrt 3 }b_{xy}^{k} } \right] $$

where the orientation of the inter-nuclear vector in the MF is described by

4a $$ b_{mn}^{k} = \left\langle {\frac{1}{2}\left( {3\cos \beta_{m}^{k} \cos \beta_{n}^{k} - \delta_{mn} } \right)} \right\rangle . $$

The term *δ*_*mn*_ represents the Kronecker delta function, *l* is the alignment condition, and *m*, *n* = *x*, *y*, *z*.

A matrix formalism is introduced to render analysis of the RDC data in a more intuitive manner (Tolman 2002). When *K* RDCs are measured under *L* alignments, then Eq. (2) becomes

5 $$ {\mathbf{D}} = \left\langle {\mathbf{B}} \right\rangle \left\langle {\mathbf{A}} \right\rangle $$

where **D** is a *K* × *L* matrix, **B** is a *K* × 5 matrix, and **A** is a 5 × *L* matrix. In Eq. (5), the term $D_{ij}^{max}$ is included in $\left\langle {\mathbf{A}} \right\rangle$. The rows of **B** are defined by Eq. (4) and the columns of **A** are given by Eq. (3). An inherent assumption in the present analysis is that inter-nuclear dynamics are uncorrelated with the alignment process; hence the averages of $\left\langle \bf{B} \right\rangle$ and $\left\langle \bf{A} \right\rangle$ are independent of each other. This assumption can be tested with the SECONDA analysis (Hus and Brüschweiler 2002; Hus et al. 2003). When the structure of the molecule is known and RDCs for at least five linearly independent inter-nuclear vectors are measured, the matrix **B** (input from the rigid structure or random structural coordinates) and the measured RDCs are used to calculate $\left\langle \bf{A} \right\rangle$

6 $$ \left\langle {\mathbf{A}} \right\rangle = {\mathbf{B}}^{ + } {\mathbf{D}} $$

where **B** ^+^ is the pseudo-inverse of **B**. It should be noted that a single alignment tensor per alignment medium is necessary for the successful application of the following protocols. Intrinsically disordered proteins (see Bertoncini et al. 2005; Bernadó et al. 2005) and multiple domain proteins (see Bertini et al. 2004; Rodriguez-Castañeda et al. 2006) will have to be described by several alignment tensors per alignment medium and will not be amenable to the present analysis.

Each column of $\left\langle \bf{A} \right\rangle$, given by Eq. (3), can be recast into *L* symmetric 3 × 3 second rank Cartesian order tensors, $(A_{l}^{(2)} )$. These order matrices are then redefined in a principal axis system (PAS), termed the alignment frame (AF), where Eq. (1) becomes (Bax et al. 2001)

7 $$ D_{k,l}^{exp} = D_{a,l} \left[ {\left\langle {3\cos^{2} \theta_{k,l}^{AF} - 1} \right\rangle + \frac{3}{2}R_{l} \left\langle {\sin^{2} \theta_{k,l}^{AF} \cos 2\phi_{k,l}^{AF}  } \right\rangle } \right]. $$

In Eq. (7), the magnitude of the alignment tensor is $D_{a,l} = \frac{1}{2}D_{ij}^{max} *A_{zz,l}^{PAS}$, the rhombicity is $R_{l} = \frac{{2\left({A_{xx,l}^{PAS} - A_{yy,l}^{PAS}} \right)}}{{3*A_{zz,l}^{PAS}}},\,(\theta_{k,l}^{AF},\phi_{k,l}^{AF})$ are the polar angles defining the inter-nuclear vector in the AF, and $A_{(mm,l)}^{PAS}$ are the eigenvalues resulting from the diagonalization of $A_{l}^{(2)}$. From the eigenvectors $\left( {A_{mn,l}^{EV} } \right)$, the Euler angles describing the rotation of $A_{l}^{(2)}$ into the PAS are defined

8 $$ \begin{aligned} \alpha_{l} &= { \arctan }\left[ {A_{xz,l}^{EV} ,A_{yz,l}^{EV} } \right],\quad \beta_{l} = { \arccos }\left[ {A_{zz,l}^{EV} } \right],\\  \gamma_{l} &= \arctan \begin{array}{*{20}c} {\left[ { - A_{zx,l}^{EV} ,A_{zy,l}^{EV} } \right].} \\ \end{array} \end{aligned}$$

### Model free analysis

With the MFA (Meiler et al. 2001; Peti et al. 2002), the five parameters describing each alignment tensor in the PAS, $\left\{ {A_{zz}^{PAS} , R, \alpha , \beta , \gamma } \right\}_{l}$, are used to construct the **F** matrix which is needed to derive the five dynamically averaged second order spherical harmonics

9a $$ \left\langle {Y_{2,0} \left( {\theta_{k}^{MF} ,\phi_{k}^{MF} } \right)} \right\rangle = \sqrt {\frac{5}{16\pi }} \left\langle {3\cos^{2} \theta_{k}^{MF} - 1} \right\rangle $$

9b $$ \left\langle {Y_{2, \pm 1} \left( {\theta_{k}^{MF} ,\phi_{k}^{MF} } \right)} \right\rangle = \mp \sqrt {\frac{15}{8\pi }} \left\langle {e^{{ \pm i{\phi_{k}^{MF} }}} \cos \theta_{k}^{MF} \sin \theta_{k}^{MF} } \right\rangle $$

9c $$ \left\langle {Y_{2, \pm 2} \left( {\theta_{k}^{MF} ,\phi_{k}^{MF} } \right)} \right\rangle = \sqrt {\frac{15}{32\pi }} \left\langle {e^{{ \pm 2i\phi_{k}^{MF} }} \sin^{2} \theta_{k}^{MF} } \right\rangle . $$

Equation (7) can be recast in terms of dynamically averaged second order spherical harmonics

10 $$ D_{k,l}^{exp} = A_{zz,l}^{PAS} \sqrt {\frac{4\pi }{5}} \left[ {\left\langle {Y_{2,0} \left( {\theta_{k,l}^{AF} ,\phi_{k,l}^{AF} } \right)} \right\rangle + \sqrt \frac{3}{8} R_{l} \left( {\left\langle {Y_{2,2} \left( {\theta_{k,l}^{AF} ,\phi_{k,l}^{AF} } \right)} \right\rangle + \left\langle {Y_{2, - 2} \left( {\theta_{k,l}^{AF} ,\phi_{k,l}^{AF} } \right)} \right\rangle } \right) } \right] $$

where

10a $$ \left\langle {Y_{2,0} \left( {\theta_{k,l}^{AF} ,\phi_{k,l}^{AF} } \right)} \right\rangle = \sqrt {\frac{5}{16\pi }} \left\langle {3\cos^{2} \theta_{k,l}^{AF} - 1} \right\rangle $$

10b $$ \left\langle {Y_{2,2} \left( {\theta_{k,l}^{AF} ,\phi_{k,l}^{AF} } \right)} \right\rangle + \left\langle {Y_{2, - 2} \left( {\theta_{k,l}^{AF} ,\phi_{k,l}^{AF} } \right)} \right\rangle = 2\sqrt {\frac{15}{32\pi }} \left\langle {\sin^{2} \theta_{k,l}^{AF} \cos 2\phi_{k,l}^{AF} } \right\rangle . $$

The **F** matrix relates the measured RDCs to the spherical harmonics defined in the MF by a Wigner rotation from the MF to the AF

11 $$ \frac{{D_{k,l}^{exp} }}{{A_{zz,l}^{PAS} }} = \mathop \sum \limits_{M = - 2}^{2} F_{l,M} \left\langle {Y_{2,M} (\theta_{k}^{MF} ,\phi_{k}^{MF} )} \right\rangle $$

with

12 $$ \begin{aligned} F_{l,M} & = \sqrt {\frac{4\pi }{5}} \left[ {D_{M0}^{2} (\alpha_{l} ,\beta_{l} ,\gamma_{l} ) + \sqrt \frac{3}{8} R_{l} \left( {D_{M2}^{2} (\alpha_{l} ,\beta_{l} ,\gamma_{l} ) + D_{M - 2}^{2} (\alpha_{l} ,\beta_{l} ,\gamma_{l} )} \right) } \right] \\ & = \sqrt {\frac{4\pi }{5}} \left[ {e^{{ - iM\alpha_{l} }} d_{M0}^{2} (\beta_{l} ) + \sqrt \frac{3}{8} R_{l} \left( {e^{{ - iM\alpha_{l} }} d_{M2}^{2} (\beta_{l} )e^{{ - i2\gamma_{l} }} + e^{{ - iM\alpha_{l} }} d_{M - 2}^{2} (\beta_{l} )e^{{i2\gamma_{l} }} } \right) } \right]. \\ \end{aligned} $$

In analogy to the component definition from Eq. (5), **Y** is a *K* × 5 matrix containing the dynamically averaged spherical harmonics in the MF and **F** is a 5 × *L* matrix containing the alignment tensor information. The $\left\langle{\mathbf{Y}}\right\rangle_{\mathbf{refined}}$ matrix is determined in direct correspondence to Eq. (6)

13 $$ \left\langle {\mathbf{Y}} \right\rangle_{{{\mathbf{refined}}}} = {\mathbf{D}}_{{{\mathbf{normalized}}}} \left\langle {\mathbf{F}} \right\rangle^{ + } . $$

Here, ${\mathbf{D}}_{{{\mathbf{normalized}}}}$ represents $\frac{D_{k,l}^{exp}}{A_{zz,l}^{PAS}}$ in order to normalize the contributions of each alignment condition to the calculation of refined structural coordinates. Each row of $\left\langle{\mathbf{Y}}\right\rangle_{\mathbf{refined}}$ is used to determine $S_{RDC,k}^{2}$

14 $$  S_{{RDC,k}}^{2}  = \frac{{4\pi }}{5}\sum\limits_{{M =  - 2}}^{2} {\left\langle {Y_{{2,M}} \left( {\theta _{k}^{{MF}} ,\phi _{k}^{{MF}} } \right)} \right\rangle } \left\langle {Y_{{2,M}}^{*} \left( {\theta _{k}^{{MF}} ,\phi _{k}^{{MF}} } \right)} \right\rangle .  $$

From the dynamically averaged spherical harmonics, the dynamically averaged orientations for each inter-nuclear vector, $\left( {\theta_{avg,k}^{MF} , \phi_{avg,k}^{MF} } \right)$, can be obtained. Maximizing $\left\langle {Y_{ 2,0} \left( {\theta_{k}^{VF} , \phi_{k}^{VF} } \right)} \right\rangle$ places the *z* axis of the vector’s axis system, termed the vector frame (VF), in the center of the inter-nuclear vector’s orientational distribution,

15 $$ \begin{aligned} { \hbox{max} }\left\langle {Y_{2,0} (\theta_{k}^{VF} ,\phi_{k}^{VF} )} \right\rangle & = \mathop \sum \limits_{M = - 2}^{2} D_{M,0} \left( {\phi_{avg,k}^{MF} ,\theta_{avg,k}^{MF} ,0} \right)\left\langle {Y_{2,M} \left( {\theta_{k}^{MF} ,\phi_{k}^{MF} } \right)} \right\rangle \\ & = \sqrt {\frac{4\pi }{5}} \sum\limits_{M = - 2}^{2} {Y_{2, - M} \left( {\theta_{avg,k}^{MF} ,\varphi_{avg,k}^{MF} } \right)\left\langle {Y_{2,M} (\theta_{k}^{MF} ,\phi_{k}^{MF} )} \right\rangle } . \\ \end{aligned} $$

The terms $\left\langle {Y_{ 2, \pm 1} \left( {\theta_{k}^{MF} , \phi_{k}^{MF} } \right)} \right\rangle$ vanish in the VF and $\left\langle {Y_{ 2, \pm 2} (\theta_{k}^{MF} , \phi_{k}^{MF} )} \right\rangle$ possesses information on the amplitude of anisotropy, *η*_*k*_, and the orientation of anisotropic motions, $\phi_{k}^{\prime}$

16 $$ \eta_{k} = \sqrt {\frac{{\mathop \sum \nolimits_{M = - 2,2} \left\langle {Y_{2,M} (\theta_{k}^{VF} ,\phi_{k}^{VF} )} \right\rangle \left\langle {Y_{2, - M} (\theta_{k}^{VF} ,\phi_{k}^{VF} )} \right\rangle }}{{\mathop \sum \nolimits_{M = - 2}^{2} \left\langle {Y_{2,M} (\theta_{k}^{VF} ,\phi_{k}^{VF} )} \right\rangle \left\langle {Y_{2, - M} (\theta_{k}^{VF} ,\phi_{k}^{VF} )} \right\rangle }}} $$

17 $$ \phi_{k}^{'} = \frac{1}{2}\arctan \frac{{\left\langle {Y_{2,2} (\theta_{k}^{VF} ,\phi_{k}^{VF} )} \right\rangle - \left\langle {Y_{2, - 2} (\theta_{k}^{VF} ,\phi_{k}^{VF} )} \right\rangle }}{{i\left( {\left\langle {Y_{2,2} \left( {\theta_{k}^{VF} ,\phi_{k}^{VF} } \right)} \right\rangle + \left\langle {Y_{2, - 2} \left( {\theta_{k}^{VF} ,\phi_{k}^{VF} } \right)} \right\rangle } \right)}}. $$

It should be noted that $S_{RDC,k}^{ 2}$ is the same in any frame, thus

18 $$ S_{RDC,k}^{2} = \frac{4\pi }{5}\mathop \sum \limits_{M = - 2}^{2} \left\langle {Y_{2,M} (\theta_{k}^{VF} ,\phi_{k}^{VF} )} \right\rangle \left\langle {Y_{2,M}^{*} (\theta_{k}^{VF} ,\phi_{k}^{VF} )} \right\rangle , $$

which is equivalent to Eq. (14).

### Standard tensorial analysis

Recalling Eq. (4), the following relationships are established in order to construct $B_{k}^{(2)}$ (Snyder 1965)

19a $$ b_{zz,k} = \sqrt {\frac{4\pi }{5}} \left\langle {Y_{2,0} (\theta_{k}^{MF} ,\phi_{k}^{MF} )} \right\rangle $$

19b $$\begin{aligned} \frac{1}{\sqrt 3 }\left( {b_{xx,k} - b_{yy,k} } \right) &= \sqrt \frac{1}{2} \sqrt {\frac{4\pi }{5}} \left[ \left\langle {Y_{2, - 2} \left( {\theta_{k}^{MF} ,\phi_{k}^{MF} } \right)} \right\rangle \right.\\ &\quad  +\left. \left\langle {Y_{2,2} \left( {\theta_{k}^{MF} ,\phi_{k}^{MF} } \right)} \right\rangle  \right] \end{aligned} $$

19c $$ \begin{aligned} \frac{2}{\sqrt 3 }b_{xz,k} &= \sqrt \frac{1}{2} \sqrt {\frac{4\pi }{5}} \left[ \left\langle {Y_{2, - 1} \left( {\theta_{k}^{MF} ,\phi_{k}^{MF} } \right)} \right\rangle \right.\\ &\quad -\left. \left\langle {Y_{2,1} \left( {\theta_{k}^{MF} ,\phi_{k}^{MF} } \right)} \right\rangle \right]\end{aligned} $$

19d $$\begin{aligned} \frac{2}{\sqrt 3 }b_{yz,k} &= i\sqrt \frac{1}{2} \sqrt {\frac{4\pi }{5}} \left[ \left\langle {Y_{2, - 1} \left( {\theta_{k}^{MF} ,\phi_{k}^{MF} } \right)} \right\rangle\right. \\ &\quad +\left. \left\langle {Y_{2,1} \left( {\theta_{k}^{MF} ,\phi_{k}^{MF} } \right)} \right\rangle  \right] \end{aligned} $$

19e $$ \frac{2}{\sqrt 3 }b_{xy,k} = i\sqrt \frac{1}{2} \sqrt {\frac{4\pi }{5}} \left[ {\left\langle {Y_{2, - 2} \left( {\theta_{k}^{MF} ,\phi_{k}^{MF} } \right)} \right\rangle - \left\langle {Y_{2,2} \left( {\theta_{k}^{MF} ,\phi_{k}^{MF} } \right)} \right\rangle } \right]. $$

The resulting eigenvalues ($$ B_{mm,k}^{PAS} ) $$ contain the dynamic information for each vector $$ \left( {S_{RDC,k}^{ 2} , \eta_{k} } \right) $$, while the eigenvectors, $$ \left( {B_{mn,k}^{EV} } \right) $$, encompass the bond orientations $$ \left({\theta_{k}^{MF}, \phi_{k}^{MF}}\right) $$ and the direction of the anisotropic local motion $$ \left( {\phi_{k}^{'} } \right) $$. The following equations detail how the dynamic parameters are calculated from $$ B_{mm,k}^{PAS} $$. The Saupe order parameters are defined as

20a $$ S_{0,k}^{2} = B_{zz,k}^{PAS} = \sqrt {\frac{4\pi }{5}} \left\langle {Y_{2,0} (\theta_{k}^{PAS} ,\phi_{k}^{PAS} )} \right\rangle $$

20b $$ S_{2,k}^{2} = \sqrt \frac{2}{3} (B_{xx,k}^{PAS} - B_{yy,k}^{PAS} ) = \sqrt {\frac{4\pi }{5}} \left[ {\left\langle {Y_{2,2} \left( {\theta_{k}^{PAS} ,\phi_{k}^{PAS} } \right)} \right\rangle + \left\langle {Y_{2, - 2} \left( {\theta_{k}^{PAS} ,\phi_{k}^{PAS} } \right)} \right\rangle } \right]. $$

21 $$ S_{RDC,k}^{2} = \left( {B_{zz,k}^{PAS} } \right)^{2} + \frac{1}{3}\left( {B_{xx,k}^{PAS} - B_{yy,k}^{PAS} } \right)^{2} = \left( {S_{0,k}^{2} } \right)^{2} + \frac{1}{2}\left( {S_{2,k}^{2} } \right)^{2} $$

22 $$ \eta_{k}^{PAS} = \sqrt {\frac{{\frac{1}{3}\left( {B_{xx,k}^{PAS} - B_{yy,k}^{PAS} } \right)^{2} }}{{S_{RDC,k}^{2} }}} = \sqrt {\frac{{\frac{1}{2}\left( {S_{2,k}^{2} } \right)^{2} }}{{S_{RDC,k}^{2} }}} . $$

For each inter-nuclear vector, $\left( {\theta_{k}^{MF} ,\phi_{k}^{MF} } \right)$ and $\phi_{k}^{\prime }$ are extracted from the transpose of the resulting $B_{mn,k}^{EV}$ matrix

23 $$ \begin{aligned} \phi_{k}^{MF} &= { \arctan }\left[ {B_{xz,k}^{EV} ,B_{yz,k}^{EV} } \right]],\quad \theta_{k}^{MF} = { \arccos }\left[ {B_{zz,k}^{EV} } \right],\\ \phi_{k}^{\prime } &= { \arctan }\left[ { - B_{zx,k}^{EV} ,B_{zy,k}^{EV} } \right].\end{aligned} $$

### Direct interpretation of dipolar couplings

With DIDC, once $\left\langle{\mathbf{A}}\right\rangle$ is determined from Eq. (6), $\left\langle {\mathbf{A}} \right\rangle$ is used to directly calculate a new set of dynamically averaged coordinates, ${\mathbf{B}}_{{{\mathbf{refined}}}}$, without extracting each set of $\left\{ {A_{zz}^{PAS} ,R,\alpha ,\beta ,\gamma } \right\}_{l}$, according to

24 $$ \left\langle {\mathbf{B}} \right\rangle_{{{\mathbf{refined}}}} = {\mathbf{D}}\left\langle {\mathbf{A}} \right\rangle^{ + }\,+\,{\mathbf{B}}\left[ {1 - \left\langle {\mathbf{A}} \right\rangle \left\langle {\mathbf{A}} \right\rangle^{ + } } \right]. $$

This formula leaves the information for $\left\langle{\mathbf{A}}\right\rangle$ in the MF. It should be noted that the previous implementations of DIDC did not scale the RDCs by $A_{zz,l}^{PAS}$ as in the MFA (see Eq. 13), which is necessary to normalize the contributions of each alignment condition for the calculation of refined structural coordinates. Therefore, we have modified Eq. (24) as follows

25 $$ \left\langle {\mathbf{B}} \right\rangle_{{{\mathbf{refined}}}} = {\mathbf{D}}_{{{\mathbf{normalized}}}} \left\langle {\mathbf{A}} \right\rangle_{{{\mathbf{normalized}}}}^{ + }\,+\,{\mathbf{B}}\left[ {1 - \left\langle {\mathbf{A}} \right\rangle \left\langle {\mathbf{A}} \right\rangle^{ + } } \right], $$

where ${\mathbf{D}}_{{{\mathbf{normalized}}}}$ and $\left\langle {\mathbf{A}} \right\rangle_{{{\mathbf{normalized}}}}$ represent the RDCs and alignment tensors divided by $A_{zz,l}^{PAS}$.

As described by Tolman, the first term in Eqs. (24) and (25) encompasses the contribution of the measured RDCs to determining $\left\langle {\mathbf{B}} \right\rangle_{{{\mathbf{refined}}}}$ (Tolman 2002). When the rank of $\left\langle {\mathbf{A}} \right\rangle$ is smaller than 5, then the second term accounts for the degeneracy in the possible solutions that results from B. Otherwise, this term will equal zero for data representing more than five alignment media. With $\left\langle{\mathbf{B}}\right\rangle_{{{\mathbf{refined}}}}$, the 3 × 3 second rank Cartesian tensor, $B_{k}^{(2)}$, for each inter-nuclear vector is constructed, diagonalized into the VF, and Eqs. (21), (22), and (23) calculate each set of $\left\{ {S_{RDC}^{2} ,\eta ,\theta_{avg}^{MF} ,\phi_{avg}^{MF} ,\phi^{\prime } } \right\}_{k}$.

### ORIUM procedure

Optimized RDC-based Iterative and Unified Model-free analysis (ORIUM) is an iterative approach (see Fig. 2) and is related to but different from the SCRM and iterative DIDC approaches as discussed in this section. The scheme is summarized as follows. First, alignment tensors, $\left\langle{\mathbf{A}}\right\rangle$, are calculated with Eq. (6) and are used to determine $\left\langle{\mathbf{B}}\right\rangle_{\mathbf{refined}}$. A comparison of the effects of scaling the RDCs with $A_{zz,l}^{PAS}$ in the determination of $\left\langle{\mathbf{B}}\right\rangle_{{{\mathbf{refined}}}}$ will be presented in the Applications section below [see Eqs. (24) and (25)]. Based on $\left\langle{\mathbf{B}}\right\rangle_{{{\mathbf{refined}}}}$, the 3 × 3 symmetric Saupe matrix is constructed for the inter-nuclear vectors using expressions defined in Eqs. (19a)–(19e), and $B_{k}^{(2)}$ is put into the local PAS. Utilizing Eqs. (21), (22), and (23), each set of $\{ S_{RDC}^{ 2} , \eta , \theta^{MF} , \phi^{MF} , \phi^{\prime } \}_{k}$ is extracted. These refined angles (*θ*_*k*_^*MF*^, *ϕ*_*k*_^*MF*^) are used as input for the next cycle of ORIUM. The cycle is finished when the convergence of order parameter is achieved using the relationship

26 $$ \frac{1}{K}\mathop \sum \limits_{k = 1}^{K} \left| {S_{RDC,k}^{2} \left( r \right) - S_{RDC,k}^{2} \left( {r - 1} \right)} \right| \le 0.0001 $$

where *r* is a cycle of iteration.

The ORIUM approach differs from the SCRM method as follows. There is a minor difference: with SCRM, the inter-nuclear vector coordinates are defined in terms of spherical harmonics, while ORIUM utilizes Cartesian coordinates. The relationship between the spherical harmonics and the Cartesian coordinates are give by Eqs. (19a)–(19e). A key difference is that SCRM requires the five alignment tensor parameters $\left\{ {A_{zz}^{PAS},R,\alpha ,\beta ,\gamma } \right\}_{l}$ to construct the **F** matrix for the determination of $\left\langle{\mathbf{Y}}\right\rangle_{{{\mathbf{refined}}}}$. DIDC and ORIUM calculate $\left\langle{\mathbf{B}}\right\rangle_{{{\mathbf{refined}}}}$ directly from $\left\langle{\mathbf{A}}\right\rangle_{{{\mathbf{normalized}}}}$. Finally, SCRM maximizes $\left\langle {Y_{ 2,0} (\theta_{k}^{VF} , \phi_{k}^{VF} )} \right\rangle$, whereas ORIUM places $B_{k}^{(2)}$ into a local axis system by diagonalization of the symmetric 3 × 3 second rank Cartesian tensor.

There are three key differences between ORIUM and the iterative DIDC approach. First, a grid search is implemented with the iterative DIDC scheme which minimizes the difference between the vector’s coordinates obtained from $\left\langle{\mathbf{B}}\right\rangle_{{{\mathbf{refined}}}}$ and an exhaustive list of $(\theta ,\phi )$ combinations to find dynamically averaged coordinates. As stated above, ORIUM diagonalizes $B_{k}^{(2)}$ into a local axis system in order to extract this information. The second key difference is that with the iterative DIDC scheme each inter-nuclear vector is constrained to be rigid $\left( {S_{RDC}^{ 2} = 1} \right)$. Only during the final iterative run is the $S_{RDC}^{ 2} = 1$ constraint removed. ORIUM never constrains the dynamics of the inter-nuclear vectors during the iterative procedure. A final divergence between the two procedures is how flexible inter-nuclear vectors are removed from the calculation of the alignment tensors. In the iterative DIDC procedure, RDC data for an individual inter-nuclear vector is removed from the calculation of the alignment tensors if the error in the experimental and back-calculated RDCs is greater than a factor of 2. The calculation is restarted and RDC data for the next inter-nuclear vector is once again removed from the calculation if the deviation is greater than a factor of 2. This procedure is repeated until all inter-nuclear vectors fulfill the threshold for the error in experimental and back-calculated RDCs. At this point, the $S_{RDC}^{2} = 1$ constraint is removed and a final iteration is performed. ORIUM removes the most flexible residues $S_{RDC}^{ 2} \le 0. 9 5$, after Eq. (26) has been satisfied (see below) and then ORIUM is restarted until Eq. (26) is once again fulfilled.

As with the RDC-based model free analysis, the fundamental assumption is that the internal protein dynamics for each inter-nuclear vector is uncorrelated with fluctuations with the alignment tensor. Thus, a single average alignment tensor can be utilized for each medium. Molecular dynamics simulations indicate that this assumption is true for secondary structural elements, however $\left\langle {\mathbf{B}} \right\rangle$ and $\left\langle {\mathbf{A}} \right\rangle$ dynamics may be correlated for the most mobile regions of a protein (Louhivuori et al. 2006; Salvatella et al. 2008). To circumvent this potential inseparability of mobile inter-nuclear vectors and the alignment tensor fluctuations, the approach outlined in the SCRM procedure is followed (Lakomek et al. 2008). After convergence is achieved with Eq. (26), the residues that are the most mobile, as determined by fulfilling the relationship $S_{RDC}^{2} \le 0.95$, are removed from the $\left\langle {\mathbf{A}} \right\rangle$ calculation and ORIUM is restarted with $\left\langle {\mathbf{B}} \right\rangle_{{{\mathbf{refined}}}}$ from the previous iteration until Eq. (26) is once again satisfied.

The validity of ORIUM was accessed with synthetic RDC data containing a measurement error (0.3 Hz) for the 36 alignment media, which was generated using the RDC refined ubiquitin ensemble ERNST (PDB:2KOX) (Fenwick et al. 2011). The corresponding dynamic parameters ($S_{RDC}^{2}$ and *η*) were also calculated using the same ensemble. Using these synthetic RDC data, ORIUM was conducted and the resulting dynamic parameters have been compared with those calculated from the ensemble. The Pearson correlations of $S_{RDC}^{2}$ and *η* are 0.97 and 0.93, respectively.

It should be noted that the local PAS differs from the VF when $B_{zz,k}^{{}}$ is a negative value, although the local PAS is usually the VF. In this case, the averaged vector orientation is actually orthogonal to the *z* axis of PAS. This issue can be alleviated by choosing a new axis system referred to the vector frame system (VFS), with eigenvalues ordered $B_{zz,k} \ge B_{xx,k} \ge B_{yy,k}$ instead of $| {B_{zz,k}^{{}} }| \ge | {B_{xx,k}^{{}} }| \ge | {B_{yy,k}^{{}} }|$. It should also be noted that $\eta$ from the VFS and the PAS can be significantly different in the case that $B_{zz,k}^{{}}$ has a negative value. ORIUM utilizes the VFS after removal of residues with $S_{RDC}^{2} \le 0.95$ to obtain dynamically averaged angles of the bond vector distribution.

### Determination of $S_{RDC}^{2}$ scaling factor: $S_{overall}$

An inherent complication when calculating $S_{RDC}^{2}$ from experimental RDCs is that dynamic averaging will reduce the actual magnitude of $A_{zz,l}^{PAS}$ or $D_{a,l}$. This reduced magnitude will result in some $S_{RDC}^{2}$ parameters over 1, and thus $S_{RDC}^{2}$ can only be considered as relative gauge of the actual amplitudes of motion, defined as $S_{RDC,unscaled}^{2}$ (Lakomek et al. 2006; Meiler et al. 2001). It should be noted that the alignment tensor parameters $\left\{ {R,\alpha ,\beta ,\gamma } \right\}_{l}$ are unaffected by the reduction in the magnitude of $A_{zz,l}^{PAS}$ or $D_{a,l}$. Two avenues to circumvent this complication have been developed. Either all the order parameters are scaled relative to the largest $S_{RDC, unscaled}^{2}$ leaving one order parameter equal to one (iterative DIDC approach) (Tolman 2002; Yao et al. 2008), or $S_{RDC, unscaled}^{2}$ is scaled relative to the Lipari-Szabo order parameters ($S_{LS}^{2}$) calculated for each residue (MFA/SCRM approach) (Lakomek et al. 2006, 2008). The problem with the first approach is that the resulting $S_{RDC}^{2}$ parameters will underestimate the amplitude of motion for each inter-nuclear vector. Overestimation can only occur if the largest $S_{RDC, unscaled}^{2}$ parameter has a large experimental error, leading to an artificially greater value for this parameter than in reality. Sub- and supra-τ_c_ motion happening for each vector equally will not be picked up by this approach, underestimating the motion except for the mentioned case. As for the second approach, $S_{LS}^{2}$ are required which may not be available for the vectors being analyzed. While this approach has been successfully applied, it may also underestimate motion since a general supra-τ_c_ motion affecting all the nuclei will not be picked up by this approach. Comparison of the $S_{overall}$ derived in Lakomek et al. 2008 and Lange et al. 2008 with the average order parameter from solid state data (Schanda et al. 2010) shows that the solid state NMR derived average order parameter is smaller than the one derived by this second approach suggesting that supra-τ_c_ motion affecting all nuclei is seen by solid state NMR but not the $S_{LS}^{2}$ versus $S_{RDC}^{2}$ approach.

Here, we present a new method for determining $S_{overall}$ without the requirement of additional information, such as $S_{LS}^{2}$, which may not be available for the inter-nuclear vectors under investigation. The scaling procedure separates an inter-nuclear vector’s motion into its principal axes in Cartesian space and leads to parameters that have a more straightforward physical interpretation. The inter-nuclear vector’s motional variance is directly related to the resulting eigenvalues calculated from diagonalization of $B_{k}^{(2)}$ into a local axis system. The methodology outlined below exploits the fact that variance cannot be negative by definition. Therefore, a uniform scaling parameter, $S_{overall}$, is necessary to insure that the variance for each inter-nuclear vector about each of the three principal axes is positive. In the following, we present a brief outline for the derivation of bond vector motional variance for the determination of $S_{overall}$.

For each vector, the following relationships between the dynamically averaged Eigenvalues and the unit vector coordinates (x, y, z) within the VF, as shown in Eq. (4a), are as follows

27 $$ B_{zz} = \frac{{3\left\langle {z^{2} } \right\rangle - 1}}{2},\quad B_{xx} = \frac{{3\left\langle {x^{2} } \right\rangle - 1}}{2},\quad B_{yy} = \frac{{3\left\langle {y^{2} } \right\rangle - 1}}{2}. $$

The normalization condition sets $x^{2} + y^{2} + z^{2} = 1$, which also implies $\left\langle {x^{2} } \right\rangle + \left\langle {y^{2} } \right\rangle + \left\langle {z^{2} } \right\rangle = 1$. Therefore, $B_{zz}$ can be recast as

28 $$ B_{zz} = \frac{{2 - 3\left\langle {x^{2} } \right\rangle - 3\left\langle {y^{2} } \right\rangle }}{2}. $$

Utilizing the definitions of $S_{RDC}^{2}$ and *η* [Eqs. (21), and (22)], we can now reformulate $S_{RDC}^{2}$ and *η* in terms of the Cartesian coordinates defined within the VF

29 $$ S_{RDC}^{2} = 1 - 3\left\langle {x^{2} } \right\rangle + 3\left\langle {x^{2} } \right\rangle^{2} - 3\left\langle {y^{2} } \right\rangle + 3\left\langle {x^{2} } \right\rangle \left\langle {y^{2} } \right\rangle + 3\left\langle {y^{2} } \right\rangle^{2} $$

30 $$ \eta = \frac{{\sqrt 3 \left( {\left\langle {x^{2} } \right\rangle - \left\langle {y^{2} } \right\rangle } \right)}}{{2\sqrt {S_{RDC}^{2} } }}. $$

The definition of variance is $\sigma_{k}^{2} = \left\langle {\left( {k - \overline{k} } \right)^{2} } \right\rangle$, where *k* = x, y. Therefore, $\sigma_{k}^{2}$ can be substituted for $\left\langle {k^{2} } \right\rangle$. Now, $S_{RDC}^{2}$ and *η* are defined in terms of variance

31 $$ S_{RDC}^{2} = 1 - 3\sigma_{x}^{2} + 3\left( {\sigma_{x}^{2} } \right)^{2} - 3\sigma_{y}^{2} + 3\sigma_{x}^{2} \sigma_{y}^{2} + 3\left( {\sigma_{y}^{2} } \right)^{2} $$

32 $$ \eta = \frac{{\sqrt 3 (\sigma_{x}^{2} - \sigma_{y}^{2} )}}{{2\sqrt {S_{RDC}^{2} } }}. $$

Solving the system of equations gives the inverse relationships

33 $$ \sigma_{x}^{2} = \frac{{1 - \sqrt {\left( {1 - \eta^{2} } \right)S_{RDC}^{2} } + \eta \sqrt {3S_{RDC}^{2} } }}{3} $$

34 $$ \sigma_{y}^{2} = \frac{{1 - \sqrt {\left( {1 - \eta^{2} } \right)S_{RDC}^{2} } - \eta \sqrt {3S_{RDC}^{2} } }}{3} $$

A graphical depiction of the mapping between these parameters is shown in Figure S1. Using the relationship ($S_{RDC}^{2} = S_{overall}^{2}  S_{RDC, unscaled}^{2}$), these equations can be written as

35 $$ \sigma_{x}^{2} = \frac{{1 - S_{overall} \sqrt {\left( {1 - \eta^{2} } \right)S_{RDC,unscaled}^{2} } + S_{overall} \eta \sqrt {3S_{RDC,unscaled}^{2} } }}{3} $$

36 $$ \sigma_{y}^{2} = \frac{{1 - S_{overall} \sqrt {\left( {1 - \eta^{2} } \right)S_{RDC,unscaled}^{2} } - S_{overall} \eta \sqrt {3S_{RDC,unscaled}^{2} } }}{3} $$

Since the variance must always be positive, the axis with the least variance ($(\sigma_{y}^{2} )$ should also be positive. Thus, the following inequalities are derived relating $S_{RDC}^{2}$ and *η* to $\sigma_{y}^{2}$

37 $$ \sigma_{y}^{2} = \frac{{1 - S_{overall} \sqrt {\left( {1 - \eta^{2} } \right)S_{RDC,unscaled}^{2} } - S_{overall} \eta \sqrt {3S_{RDC,unscaled}^{2} } }}{3} \ge 0 $$

38 $$ \begin{aligned} S_{overall} &\le \frac{1}{{\sqrt {\left( {1 - \eta^{2} } \right)S_{RDC,unscaled}^{2} } + \eta \sqrt {3S_{RDC,unscaled}^{2} } }} \\ &= - \frac{1}{{2B_{yy}^{unscaled} }} = S_{overall}^{max} .\end{aligned} $$

Using Eq. (38), residue-specific $S_{overall}^{max}$ can be obtained using $S_{RDC}^{2}$ and *η*, or from the lowest eigenvalue. The eigenvalue definition of $S_{overall}^{max}$ follows directly from Eq. (27).

Since the reduction of magnitude in the alignment due to dynamic averaging is a global effect throughout all residues, the least residue-specific $S_{overall}^{max}$ may be utilized as the scaling factor, if there is no experimental error. The previous method in which all order parameters are scaled relative to the largest $S_{RDC, unscaled}^{2}$, leaving one order parameter equal to one (iterative DIDC approach) (Tolman 2002; Yao et al. 2008) is related to this new approach. If bond vector anisotropy is assumed to be axially symmetric (*η* = 0), $S_{overall}^{max}$ turns into $1/S_{RDC,unscaled}$. This is identical to scaling all inter-nuclear vectors such that the largest is 1 (Figure S1).

This scaling approach using the lowest residue-specific $S_{overall}^{max}$ may introduce a systematic bias due to the fact that experimental data contain errors. In order to alleviate the systematic bias, we used a statistical procedure accounting for the effect of experimental noise on $S_{overall}$ without any knowledge of $S_{LS}^{2}$ unlike the SCRM approach. First, scaling factors were calculated from the original data as well as datasets with noise added equivalent to the experimental error. These scaling factors were used to determine a value (which we term $S_{overall}^{95\,\% }$), below which there was a 95 % chance that the true $S_{overall}$ would fall. Given the maximum scaling factor from the original data that fulfills the constraint equation for all inter-nuclear vectors, $S_{overall}^{max}$, and corresponding set of values from noise added data (NAD), $S_{overall,NAD}^{max}$, the $S_{overall}^{95\,\% }$ value can be calculated as follows:

39 $$ S_{overall}^{95\,\% } = \frac{{S_{overall}^{max} }}{{\left\langle {S_{overall,NAD}^{max} } \right\rangle }}quantile(S_{overall,NAD}^{max} ,95\,\% ) $$

where the quantile function returns the given quantile of the set. The quantile prefactor compensates for systematic shifts resulting from the addition of experimental error. With the previous study (Lakomek et al. 2008), the determination of $S_{overall}$ was conservative in order to circumvent the chance for over-estimating the supra-τ_c_ motion, reflected in the reported $S_{RDC}^{2}$. Here, the criterion for scaling is that $\sigma_{y}^{2}$ should be positive, which possesses no time-scale bias. Yet, it should be noted that this overall order parameter is an upper limit for $S_{overall}$ since it could underestimate motion if there is a uniform sub- or supra-τ_c_ motion affecting all vectors. This is summarized in Table 1.

**Table 1:** Summary of the methods for determining $S_{overall}$

Scaling	$S_{RDC}^{2} \le 1$	$S_{RDC}^{2} \le S_{LS}^{2}$	$\sigma_{y}^{2} \ge 0$ (ORIUM)	Solid state	3D GAF cross validation
Advantage	No other data required	Sub-τ_c_ motion included	No other data required	All motion is reflected in the order parameters	No other data are required
Disadvantage	Motion in the most rigid internuclear vector leads to underestimation of $S_{overall}$	Homogeneous supra-τ_c_ motion leads to underestimation of $S_{overall}$	Homogenous sub- and supra-τ_c_ motion leads to underestimation of $S_{overall}$	Different sample than solution	Gaussian fluctuations assumed, several sets of RDCs required
Reference	Yao et al. (2008)	Lakomek et al. (2008)		Schanda et al. (2010)	Salmon et al. (2009)

## Applications

### Ubiquitin: comparison of ORIUM with SCRM

In order to compare ORIUM with the SCRM method, N–H^N^ RDCs were used from measurements performed in 36 different alignment media (D36M) for the 76-residue protein ubiquitin (see Lakomek et al. 2008 for RDCs and references therein). The X-ray structure 1UBI of ubiquitin was used as the input structure for the first cycle of ORIUM (Ramage et al. 1994). For the error estimation of each extracted set of $\left\{ {S_{RDC}^{2} ,\eta } \right\}_{k}$, 1,000 Monte Carlo simulations were performed by adding uncertainty to the RDCs drawn from a Gaussian distribution with a standard deviation given by the error in the RDC set (0.3 Hz). On a single core of an Intel Core i7-2635QM CPU, the 1,000 Monte Carlo simulations required 18 min for ORIUM versus 83 min for SCRM, where the convergence criterion was set as in the SCRM implementation (Lakomek et al. 2008). When we used a 100-fold stricter convergence criterion than SCRM (see Eq. 26), the calculation was still faster on the same CPU (74 min). Thus, ORIUM is an optimized approach for the better convergence of the dynamic parameters.

**Fig. 3:**
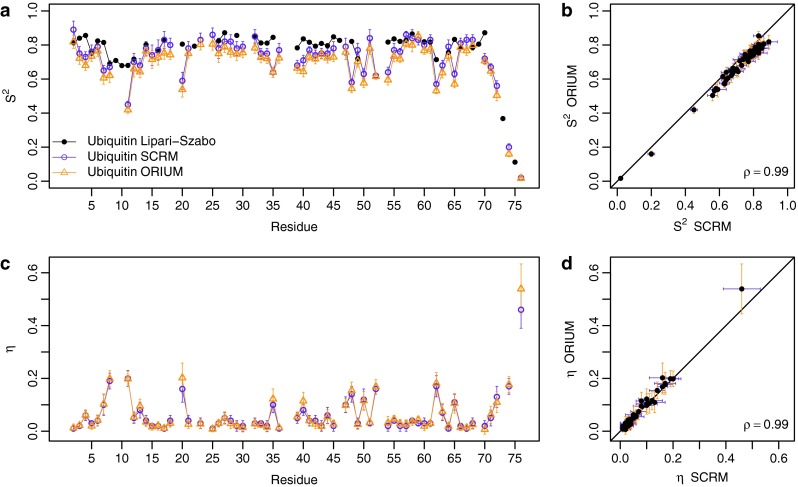
Comparison of ORIUM (*yellow*) and SCRM (*violet*) derived N–H^N^ $S_{RDC}^{2}$ and *η* parameters for ubiquitin. For both ORIUM and SCRM, the estimated error comes from 1,000 Monte Carlo simulations that add uncertainty to the RDCs drawn from a Gaussian distribution with a standard deviation given by the error in the RDC set (0.3 Hz). **a** $S_{RDC}^{2}$ plot by residue. The *black line* represents the $S_{LS}^{2}$ parameters (Chang and Tjandra 2005). **b** $S_{RDC}^{2}$ correlation plot. **c** *η* plot by residue. **d** *η* correlation plot

A comparison of the D36M RDC data set analyzed with ORIUM and the SCRM method (re-implemented in this study) is presented in Fig. 3, which shows $S_{RDC}^{2}$ and *η* determined for each residue (see Table S1 for the actual values calculated from the ORIUM analysis of the D36M data set). The correlation coefficients for the $S_{RDC}^{2}$ and *η* parameters are both 0.99, which shows that in principle both the iterative DIDC and SCRM should yield identical results. However, in the previous implementations of DIDC (Tolman 2002; Yao et al. 2008), the effect of the alignment magnitude on the angle calculation, as shown in Eqs. (24) and (25), was not recognized, leading to variations in the $S_{RDC}^{2}$ and *η* values (Figure S2).

To determine whether normalization by alignment strength produces more accurate results, we compared Q factors for each alignment condition (Figure S3). For both the standard fitting procedure and a cross validation procedure, normalization produced significantly better Q factors (with respective *p* values of 0.022 and 0.00033). In the unnormalized case without cross validation, stronger alignment conditions showed lower Q factors than weak conditions, indicating that they were contributing disproportionately to the fit. This lack of proportionality is also evident in an examination of the alignment tensors themselves. To estimate the degree to which the five dimensional alignment tensor space is uniformly sampled, condition numbers can be used, where a lower value indicates more uniform sampling (Peti et al. 2002). We checked the condition numbers of $\left\langle {\mathbf{A}} \right\rangle$ and $\left\langle {\mathbf{A}} \right\rangle_{{{\mathbf{normalized}}}}$ for ORIUM implemented with Eqs. (24) and (25), respectively. Figure S4 plots the condition number versus ORIUM iteration until Eq. (26) is satisfied. For the unnormalized alignment tensors ($\left\langle {\mathbf{A}} \right\rangle$ using Eq. 24), the condition number finishes at 8.36, while for the normalized alignment tensors ($\left\langle {\mathbf{A}} \right\rangle_{{{\mathbf{normalized}}}}$ using Eq. 25) the condition number is significantly lower finishing at 6.19. This shows that normalization of the RDCs based on $A_{zz,l}^{PAS}$ is important to adjust the contributions of strong versus weak alignments in the calculation of inter-nuclear vector orientations and dynamics. It should also be mentioned that the different values of the condition numbers do not directly indicate the reliability of the dynamics but rather the degree to which alignment space is uniformly sampled.

The slight deviation in $S_{RDC}^{2}$ between ORIUM and SCRM (Fig. 3b) originates from the $S_{overall}$, which is 0.87 from the ORIUM approach compared with the reported value of 0.89 from the SCRM report using $S_{LS}^{2}$ to calculate $S_{overall}$. Remarkably, the present approach for $S_{overall}$ determination yields scaled $S_{RDC}^{2}$ parameters that are below or within error of the $S_{LS}^{2}$ values without utilizing $S_{LS}^{2}$ in the calculation of $S_{overall}$. Due to the slightly lower $S_{overall}$ with ORIUM, the average $S_{RDC}^{2}$ for ORIUM and SCRM is 0.69 and 0.72, respectively.

In addition to starting with the 1UBI structure, the ORIUM calculation was also tested with random coil input structural coordinates and results are identical to those starting with 1UBI. This worked for ORIUM and not other procedures like SCRM because at the beginning of iteration, a sizable fraction of residues used for alignment tensor calculation had their largest eigenvalue with a negative sign. For these residues, the angle used for tensor calculation was then orthogonal to the mean angle. By the time the iteration was completed, no residues had negative maximum eigenvalues. This result shows the potential utility of ORIUM in the calculation of structural parameters from random structural input and perhaps used in the refinement of conformational ensembles. Both ideas are currently under investigation.

It is also interesting to compare the results from ORIUM, specifically in regard to the calculated $S_{overall}$ of 0.87, to a recent study examining the dynamics of ubiquitin in the microcrystalline state (Schanda et al. 2010). Here, the scaling of the solid-state order parameters ($S_{SS}^{2}$) is unnecessary since the protein is not tumbling and therefore the calculated order parameters should reflect the absolute magnitude of the amplitudes of motion for each inter-nuclear vector. The time-scale of motion embodied in $S_{SS}^{2}$ spans up to about one digit microsecond (Chevelkov et al. 2010), whereas $S_{RDC}^{2}$ encompasses motion up to about one millisecond. In principle, $S_{SS}^{2}$ is expected to be higher than $S_{RDC}^{2}$ due to the time-scale of motion embodied by the two order parameters, assuming that both the conditions for ubiquitin in microcrystalline and in solution are identical. The previously reported $S_{RDC}^{2}$ values using $S_{overall}$ of 0.89 (Lakomek et al. 2008) are on average higher than the order parameters reported for the microcrystalline state (Schanda et al. 2010). The average order parameter values of $S_{RDC}^{2}$ and $S_{SS}^{2}$ for residues 2–70 are 0.75 and 0.72, respectively. This may be due to the fact that the determination of $S_{overall}$ was done to circumvent the chance for over-estimating the supra-τ_c_ motion and thus the $S_{overall}$ was too conservative as described earlier. Figure S5 shows that with the approach presented here for the calculation of $S_{overall}$ without any time-scale bias most of the residues possess $S_{RDC}^{2}$ that are comparable or within the error of $S_{SS}^{2}$.

### GB3: comparison of ORIUM with iterative DIDC

We compared ORIUM with the iterative DIDC method. N–H^N^ and Cα–Hα RDCs were used from measurements performed on 6 mutants of the third immunoglobulin domain of protein G (GB3) aligned with Pf1 phages (Yao et al. 2008). The reported error in the N–H^N^ and Cα–Hα RDCs were 0.2 and 0.4 Hz, respectively. The NMR structure 2OED of GB3 was used as the input structure for the first cycle of ORIUM (Ulmer et al. 2003). From the SECONDA analysis of the RDC data as described in the iterative DIDC publication (see Fig. 4 in Yao et al. 2008), the following RDCs were removed from the entire analysis due to inconsistencies in the structure and dynamics for these inter-nuclear vectors over the 6 different alignment media: residues 19 and 41 for the N–H^N^ RDCs and residues 11, 25, 30, and 40 for the Cα–Hα RDCs. Error estimation followed the same protocol used for ubiquitin.

**Fig. 4:**
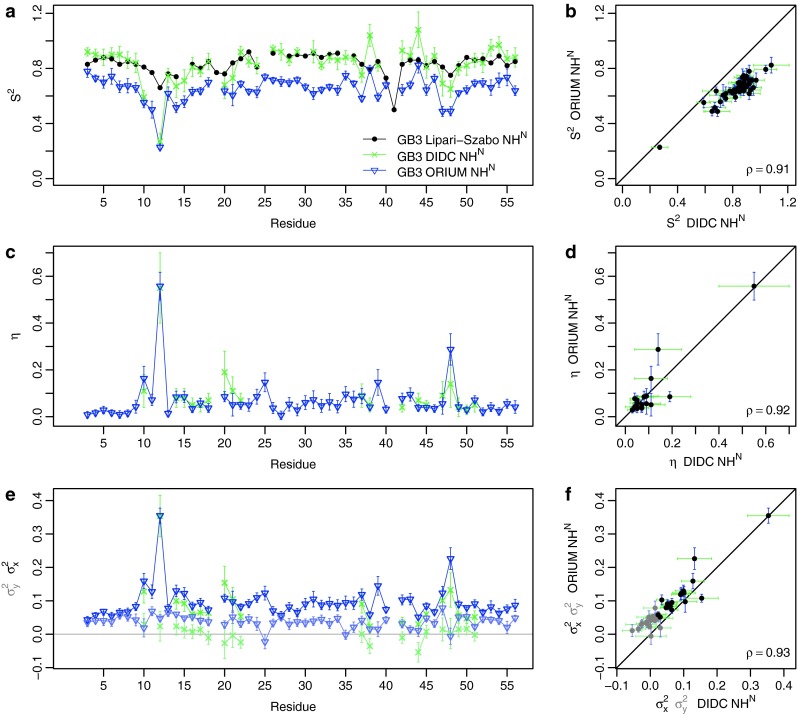
Comparison of ORIUM (*blue*) and iterative DIDC (*green*) derived N–H^N^ $S_{RDC}^{2}$ and *η* parameters for GB3. The ORIUM calculation was performed with only the N–H^N^ RDCs. For ORIUM, the estimated error results from 1,000 Monte Carlo simulations that add uncertainty to the RDCs drawn from a Gaussian distribution with a standard deviation given by the error in the experimental RDCs reported in the iterative DIDC publication of 0.2 Hz. From the iterative DIDC analysis, 100 Monte Carlo simulations were performed with an uncertainty of 0.3 Hz and *η* is determined only for residues that were not fit to an isotropic motional model (Yao et al. 2008). **a** $S_{RDC}^{2}$ plot by residue. The *solid line* represents the $S_{LS}^{2}$ parameters (Hall and Fushman 2003). **b** $S_{RDC}^{2}$ correlation plot. **c** *η* plot by residue. **d** *η* correlation plot. **e** $\sigma_{x}^{2}$ and $\sigma_{y}^{2}$ (*lighter colors*) by residue. **f** $\sigma_{x}^{2}$ and $\sigma_{y}^{2}$ (*lighter colors*) correlation plot

**Fig. 5:**
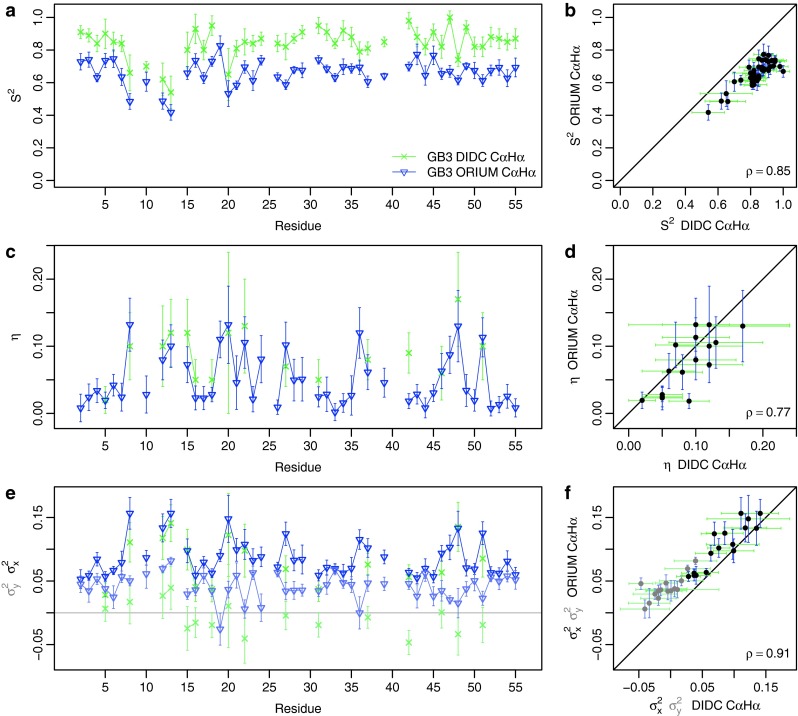
Comparison of ORIUM (*blue*) and iterative DIDC (*green*) derived Cα–Hα $S_{RDC}^{2}$ and *η* parameters for GB3. The ORIUM calculation was performed with only the Cα–Hα RDCs. For ORIUM, the estimated error results from 1,000 Monte Carlo simulations that add uncertainty to the RDCs drawn from a Gaussian distribution with a standard deviation given by the error in the experimental RDCs reported in the iterative DIDC publication of 0.4 Hz. From the iterative DIDC analysis, 100 Monte Carlo simulations were performed with an uncertainty of 0.6 Hz and *η* is determined only for residues that were not fit to an isotropic motional model (Yao et al. 2008). **a** $S_{RDC}^{2}$ plot by residue. The *solid line* represents the $S_{LS}^{2}$ parameters. **b** $S_{RDC}^{2}$ correlation plot. **c** *η* plot by residue. **d** *η* correlation plot. **e** $\sigma_{x}^{2}$ and $\sigma_{y}^{2}$ (*lighter colors*) by residue. **f** $\sigma_{x}^{2}$ and $\sigma_{y}^{2}$ (*lighter colors*) correlation plot

The ORIUM calculation was performed with only the N–H^N^ RDCs or the Cα–Hα RDCs using the actual errors in the RDC data of 0.2 and 0.4 Hz for the N–H^N^ RDCs and the Cα–Hα RDCs, respectively. The results are illustrated in Figs. 4 and 5 and compiled in Tables S2 and S3. The correlation for the N–H^N^ RDCs is 0.91 for $S_{RDC}^{2}$ and is 0.92 for *η*. As for the Cα–Hα RDCs, the correlation coefficients for $S_{RDC}^{2}$ and *η* are 0.85 and 0.77, respectively. Here, the $S_{overall}$ calculated from the ORIUM approach is 0.83 for both RDC types. This $S_{overall}$ scaling leads to an average N–H^N^ $S_{RDC}^{2}$ of 0.65 and Cα–Hα $S_{RDC}^{2}$ of 0.66. Thus, the $S_{RDC}^{2}$ values are on average 22 % lower than in the iterative DIDC publication. As shown in Figs. 4e and 5e, iterative DIDC has significantly more negative variances than ORIUM. This is primarily because the $\sigma_{y}^{2} \ge \, 0$ constraint used for ORIUM is more restrictive than the $S_{RDC}^{2} \le { 1}$ constraint used for iterative DIDC (Figure S1), making the ORIUM $S_{RDC}^{2}$ values lower than iterative DIDC.

In principle, both ORIUM and the iterative DIDC should give identical results as described earlier. The discrepancy reflected in the correlations may originate from the removal of the bias in the calculated structural and dynamic parameters due to the magnitude of alignment media [see Eqs. (24) and (25)]. In order to check this possibility, we calculated $\left\langle {\mathbf{B}} \right\rangle_{{{\mathbf{refined}}}}$ with ORIUM from the N–H^N^ RDCs and the Cα–Hα RDCs simultaneously using Eq. (24) instead of Eq. (25). For these calculations, uncertainties of 0.3 and 0.6 Hz were used for the MC analysis as done in iterative DIDC method and the Cα–Hα RDCs were scaled by a factor of 2.08^−1^ (Bax et al. 2001) (see Figures S6 and S7). Because of the small number of alignment conditions for GB3, it is not possible determine significant differences in a Q factor analysis with and without normalization. However, we checked the condition numbers of $\left\langle {\mathbf{A}} \right\rangle$ (Peti et al. 2002) for ORIUM implemented with either Eq. (24) or Eq. (25) (see Figure S8). For the unnormalized alignment tensors, the condition number finishes at 7.84, while for the normalized alignment tensors the condition numbers are again lower finishing at an average of 7.15. The correlation for the N–H^N^ RDCs is 0.94 for $S_{RDC}^{2}$ and is 0.96 for *η*. As for the Cα–Hα RDCs, the correlation coefficients for $S_{RDC}^{2}$ and *η* are 0.88 and 0.91, respectively. Although the correlations are improved, the remaining discrepancies may result from the differences in the implementation of ORIUM versus the iterative DIDC. The iterative DIDC method utilizes a grid search to find $\left( {\theta_{avg,k}^{MF} ,\phi_{avg,k}^{MF} } \right)$ for each inter-nuclear vector. During the grid search, each vector is constrained to be rigid, $(S_{RDC}^{2} = 1)$, until the final iterative run when the constraints are lifted and the dynamic parameters calculated. As in case with normalized ORIUM, unnormalized ORIUM shows lower $S_{RDC}^{2}$ values than DIDC, which results from the less restrictive DIDC constraint ($S_{RDC}^{2} \le { 1}$) allowing more negative variances (Figures S6 and S7).

## Conclusions

With ORIUM, we present a computationally efficient method for extracting structural and dynamic information for inter-nuclear vectors from RDCs unifying previously published concepts into one compact protocol. Furthermore, we demonstrate a new scheme for scaling the derived $S_{RDC}^{2}$ parameters based on variances of a single type of RDC without needing $S_{LS}^{2}$ as a constraint which constitutes an upper limit of $S_{overall}$. Dynamics occurring on time-scales slower than the rotational correlation time of proteins, encoded in RDCs, have important implications protein functionality, including enzyme catalysis, molecular recognition, and correlated motions. The concepts set forth in this paper will go far in streamlining the procedure for calculating the dynamic average orientation and associated amplitudes of motion for inter-nuclear vectors in an iterative manner as long as RDC data sets are acquired in at least five independent alignment media.

## Electronic supplementary material

Below is the link to the electronic supplementary material.

Supplementary material 1 (PDF 1992 kb)
