# Supplementary material for: ORIUM: Optimized RDC-based Iterative and Unified Model-free analysis
Source: J Biomol NMR. 2013 Sep 8;58(4):287–301. doi: 10.1007/s10858-013-9775-1 (PMC3982212; doi:10.1007/s10858-013-9775-1)
Supplement: Supplementary file 1 — Supplementary material 1 (PDF 1992 kb) [file 10858_2013_9775_MOESM1_ESM.pdf]

Supporting Information

Table S1 ORIUM derived N-H^N S^2_{RDC} and η for ubiquitin

residue	S^2_{RDC}	error	η	error	residue	S^2_{RDC}	error	η	error
1					39	0.66	0.03	0.06	0.02
2	0.82	0.04	0.01	0.01	40	0.64	0.03	0.11	0.03
3	0.72	0.04	0.02	0.01	41	0.73	0.02	0.04	0.02
4	0.68	0.03	0.06	0.02	42	0.72	0.03	0.03	0.02
5	0.73	0.03	0.02	0.01	43	0.74	0.03	0.02	0.01
6	0.76	0.03	0.04	0.02	44	0.72	0.03	0.05	0.03
7	0.61	0.04	0.10	0.04	45	0.73	0.03	0.03	0.02
8	0.62	0.04	0.20	0.03	46				
9					47	0.76	0.04	0.10	0.03
10					48	0.54	0.02	0.15	0.03
11	0.42	0.02	0.20	0.03	49	0.70	0.03	0.03	0.02
12	0.66	0.05	0.05	0.02	50	0.58	0.03	0.12	0.03
13	0.64	0.03	0.09	0.03	51	0.78	0.04	0.03	0.01
14	0.76	0.03	0.03	0.02	52	0.62	0.02	0.17	0.03
15	0.72	0.05	0.02	0.01	53				
16	0.73	0.05	0.02	0.02	54	0.59	0.03	0.03	0.02
17	0.75	0.04	0.01	0.01	55	0.73	0.03	0.05	0.01
18	0.74	0.03	0.03	0.02	56	0.72	0.02	0.03	0.02
19					57	0.81	0.02	0.03	0.02
20	0.54	0.04	0.20	0.06	58	0.80	0.04	0.04	0.01
21	0.75	0.03	0.03	0.02	59	0.85	0.03	0.04	0.01
22					60	0.77	0.02	0.02	0.02
23	0.80	0.03	0.03	0.02	61	0.78	0.03	0.03	0.01
24					62	0.53	0.02	0.18	0.04
25	0.81	0.04	0.01	0.01	63	0.64	0.03	0.07	0.03
26	0.75	0.05	0.03	0.01	64	0.73	0.02	0.02	0.01
27	0.79	0.03	0.05	0.02	65	0.57	0.02	0.11	0.04
28	0.76	0.03	0.03	0.02	66	0.79	0.03	0.01	0.01
29	0.75	0.04	0.02	0.02	67	0.77	0.03	0.01	0.01
30	0.75	0.03	0.01	0.02	68	0.80	0.03	0.03	0.02
31					69				
32	0.78	0.03	0.03	0.02	70	0.71	0.02	0.01	0.02
33	0.73	0.03	0.02	0.01	71	0.65	0.03	0.06	0.03
34	0.72	0.03	0.02	0.02	72	0.50	0.03	0.11	0.04
35	0.64	0.03	0.12	0.04	73				
36	0.72	0.03	0.01	0.01	74	0.16	0.02	0.18	0.03
37					75				
38					76	0.02	0.01	0.54	0.09

Table S2 ORIUM derived N-H[N] S^2_{RDC} and η for GB3

residue	S^2_{RDC}	error	η	error	residue	S^2_{RDC}	error	η	error
1					29	0.72	0.02	0.03	0.03
2					30	0.66	0.02	0.06	0.03
3	0.78	0.04	0.01	0.01	31	0.62	0.04	0.07	0.04
4	0.73	0.02	0.02	0.01	32	0.65	0.02	0.05	0.03
5	0.70	0.04	0.03	0.02	33	0.67	0.02	0.06	0.04
6	0.74	0.06	0.02	0.01	34	0.64	0.04	0.04	0.03
7	0.67	0.04	0.01	0.02	35	0.75	0.03	0.10	0.03
8	0.67	0.05	0.01	0.01	36	0.69	0.02	0.07	0.03
9	0.66	0.04	0.04	0.03	37	0.58	0.02	0.09	0.03
10	0.55	0.04	0.16	0.05	38	0.79	0.03	0.04	0.01
11	0.50	0.06	0.07	0.02	39	0.59	0.03	0.15	0.05
12	0.23	0.02	0.56	0.06	40	0.68	0.03	0.03	0.02
13	0.62	0.05	0.01	0.01	41				
14	0.52	0.04	0.08	0.03	42	0.64	0.04	0.08	0.02
15	0.56	0.04	0.09	0.02	43	0.68	0.03	0.10	0.03
16	0.63	0.03	0.03	0.02	44	0.82	0.06	0.04	0.01
17	0.64	0.03	0.06	0.02	45	0.64	0.04	0.04	0.01
18	0.70	0.03	0.04	0.02	46	0.73	0.03	0.04	0.02
19					47	0.49	0.04	0.06	0.04
20	0.64	0.03	0.09	0.02	48	0.49	0.03	0.29	0.07
21	0.61	0.08	0.05	0.05	49	0.62	0.02	0.04	0.02
22	0.69	0.02	0.05	0.02	50	0.64	0.03	0.03	0.01
23	0.63	0.02	0.05	0.03	51	0.69	0.03	0.07	0.02
24	0.63	0.04	0.09	0.03	52	0.69	0.03	0.02	0.02
25	0.74	0.02	0.15	0.04	53	0.66	0.06	0.04	0.02
26	0.71	0.02	0.04	0.02	54	0.71	0.05	0.02	0.02
27	0.70	0.04	0.01	0.02	55	0.73	0.06	0.06	0.02
28	0.70	0.03	0.05	0.03	56	0.64	0.04	0.04	0.03

Table S3 ORIUM derived Cα-Hα S^2_{RDC} and η for GB3

residue	S^2_{RDC}	error	η	error	residue	S^2_{RDC}	error	η	error
1					29	0.68	0.05	0.05	0.03
2	0.73	0.05	0.01	0.02	30				
3	0.74	0.05	0.02	0.02	31	0.74	0.03	0.02	0.01
4	0.63	0.03	0.03	0.02	32	0.68	0.02	0.03	0.03
5	0.74	0.04	0.02	0.01	33	0.63	0.03	0.00	0.01
6	0.75	0.05	0.04	0.02	34	0.70	0.05	0.02	0.01
7	0.63	0.05	0.02	0.02	35	0.69	0.03	0.03	0.03
8	0.48	0.05	0.13	0.04	36	0.69	0.04	0.12	0.04
9					37	0.61	0.04	0.06	0.03
10	0.61	0.06	0.03	0.03	38				
11					39	0.64	0.02	0.05	0.02
12	0.49	0.05	0.08	0.03	40				
13	0.42	0.05	0.10	0.03	41				
14					42	0.70	0.03	0.02	0.01
15	0.66	0.04	0.07	0.03	43	0.77	0.06	0.03	0.01
16	0.74	0.04	0.02	0.02	44	0.64	0.06	0.01	0.01
17	0.63	0.03	0.02	0.02	45	0.77	0.06	0.03	0.01
18	0.73	0.03	0.03	0.01	46	0.65	0.03	0.06	0.03
19	0.83	0.06	0.11	0.03	47	0.67	0.04	0.09	0.03
20	0.53	0.08	0.13	0.06	48	0.62	0.03	0.13	0.05
21	0.58	0.03	0.05	0.04	49	0.70	0.02	0.03	0.02
22	0.70	0.04	0.11	0.04	50	0.67	0.05	0.02	0.02
23	0.61	0.06	0.02	0.02	51	0.61	0.04	0.11	0.03
24	0.74	0.03	0.08	0.04	52	0.67	0.03	0.01	0.01
25					53	0.69	0.05	0.01	0.01
26	0.63	0.03	0.01	0.01	54	0.63	0.05	0.03	0.02
27	0.59	0.03	0.10	0.03	55	0.69	0.06	0.01	0.01
28	0.68	0.02	0.05	0.03	56				

Table S4

ORIUM Dataset	$S_{overall}^{95\%}$	Average Confidence Level
Ubiquitin N-H^N	0.87	0.83
GB3 NH	0.83	0.82
GB3 Ca-Hα	0.83	0.83

Average confidence levels, $\langle 1-\alpha \rangle$, in the $S_{overall}^{95\%}$ value were estimated using the statistical framework described in Lakomek 2008. In the nomenclature used for this manuscript, it can be calculated with the following equation for all combinations of *i* and *j*:

(S1) $$\langle 1-\alpha \rangle = P\left(\frac{1}{{S_{overall,NAD,i}^{max}}^2} - \frac{1}{{S_{overall,NAD,j}^{max}}^2} \leq \frac{1}{{S_{overall}^{max}}^2} - \frac{1}{{S_{overall}^{95\%}}^2}\right)$$

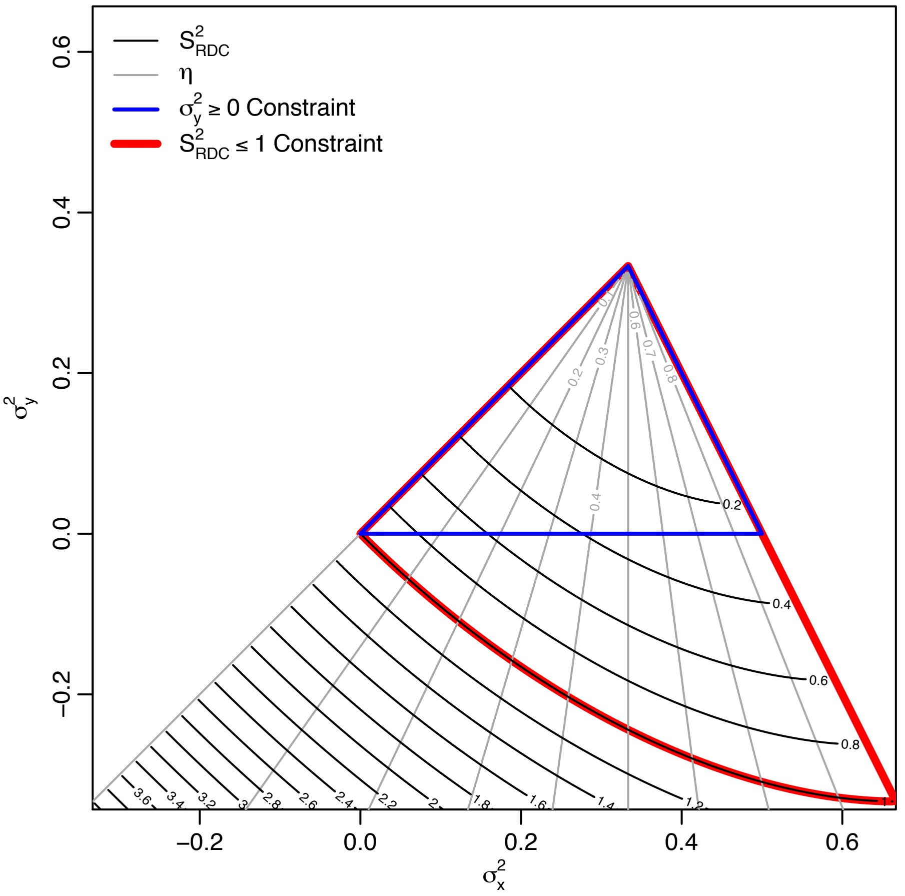

Figure S1: Graphical depiction of the mapping between the $\{\sigma_x^2, \sigma_y^2\}_k$ and $\{S_{RDC}^2, \eta\}_k$ parameter sets. Contours are only shown for the region where the $B_{zz,k} \geq B_{xx,k} \geq B_{yy,k}$ vector frame system (VFS) condition is true. S_{RDC}^2 contour lines are shown in black and increase in value the further they are from the $\left\{\frac{1}{3}, \frac{1}{3}\right\}$ coordinate. η contour lines are shown in gray and emanate from the $\left\{\frac{1}{3}, \frac{1}{3}\right\}$ coordinate. Scaling by $S_{overall}$ moves points along the η contour lines either towards or away from the $\left\{\frac{1}{3}, \frac{1}{3}\right\}$ coordinate. The region allowed by the $\sigma_y^2 \geq 0$ constraint is shown in blue. The region allowed by the $S_{RDC}^2 \leq 1$ constraint is shown in red.

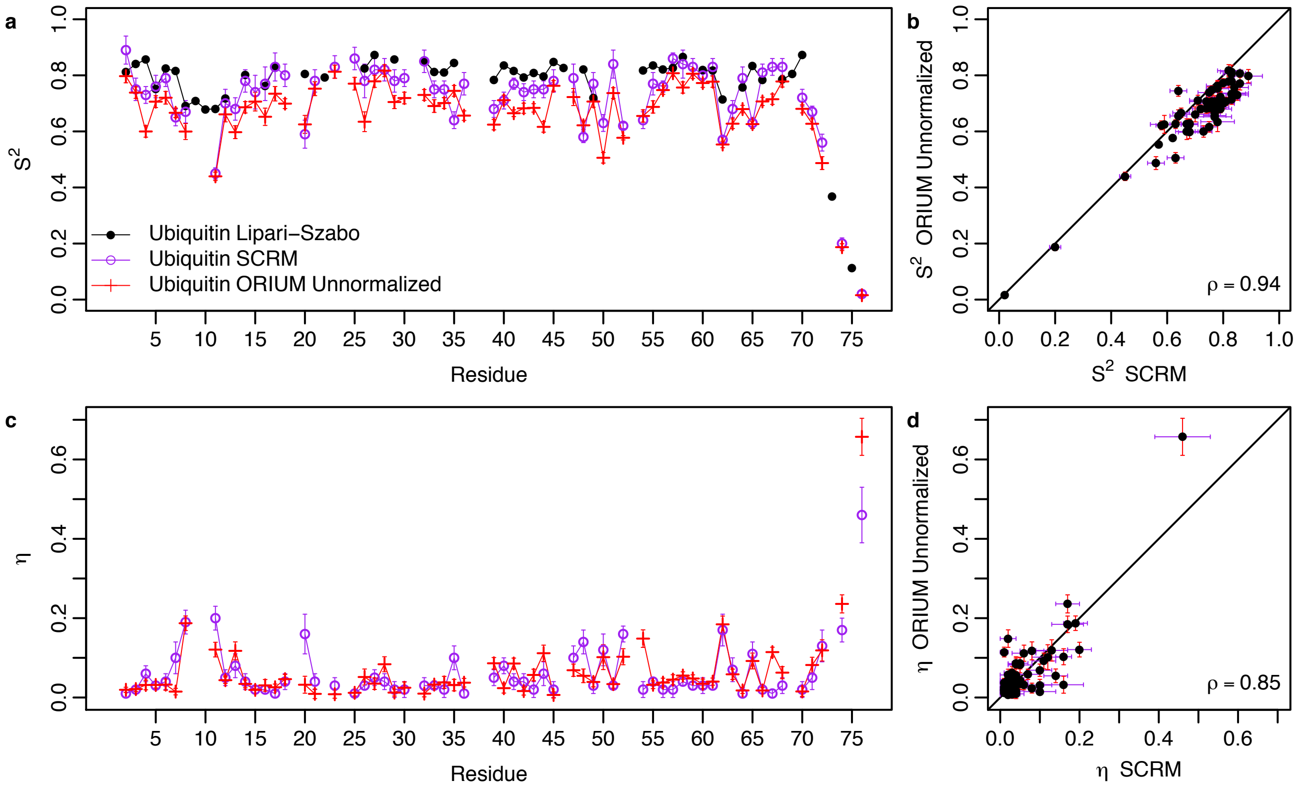

Figure S2: Comparison of ORIUM using equation (24) (red) and SCRM (violet) derived N-H^N S^2_{RDC} and η parameters for ubiquitin. For both ORIUM and SCRM, the estimated error comes from 1000 Monte Carlo simulations that add uncertainty to the RDCs drawn from a Gaussian distribution with a standard deviation given by the error in the RDC set (0.3 Hz). (a): S^2_{RDC} plot by residue. The black line represents the S^2_{LS} parameters (Chang and Tjandra 2005). (b): S^2_{RDC} correlation plot. (c): η plot by residue. (d): η correlation plot.

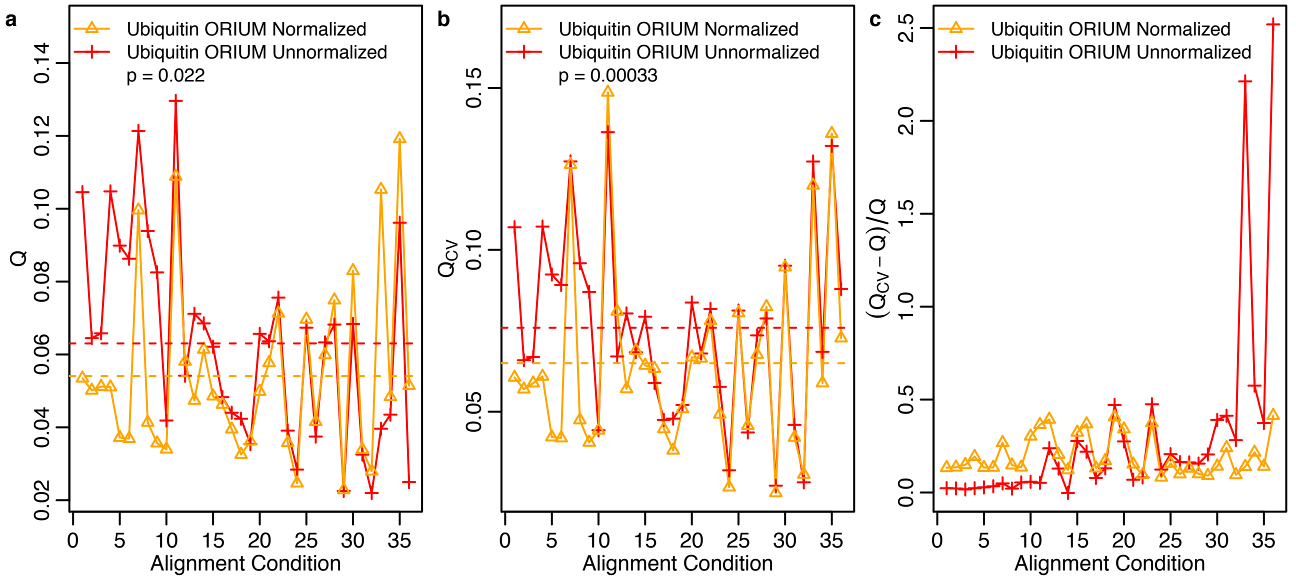

Figure S3: Comparison of Q factors for ubiquitin RDCs calculated with normalized (orange) vs. unnormalized (red) ORIUM. Alignment conditions are sorted by increasing alignment tensor strength, $|D_{a,l}|$. (a): Q factors are from the fits shown in Figures 3 and S2. For unnormalized ORIUM, there is a recognizable trend where strong alignment tensors have on average lower Q factors, indicating that they are contributing disproportionately to the fit. No such trend is observed for normalized ORIUM. Mean Q factors (dashed lines) show that normalized ORIUM is significantly more accurate than unnormalized, with the p-value taken from a paired Wilcoxon test. The mean Q factor for normalized ORIUM is comparable to the previously reported D36M Qdyn (Lakomek 2008), which is 0.052 when an applied $1/\sqrt{2}$ normalization was factor removed (personal communication, Nils Lakomek) (b): Cross-validated Q factors (Q_{CV}) were calculated using 36 separate ORIUM runs, where in each, RDCs from the given alignment condition were excluded from all $\langle \mathbf{B} \rangle_{\mathbf{refined}}$ calculations. In that case, there is no overall correlation between tensor strength and Q factor for either normalized or unnormalized. In the cross-validated case, the increase in accuracy is more significant, with 28 out of 36 alignment conditions showing better Q factors. (c): The difference between Q_{CV} and Q as a fraction of Q shows that in the unnormalized case, weak alignment tensors show relatively little reduction in accuracy upon cross validation and strong tensors show large reductions in accuracy. There is no trend for the normalized case.

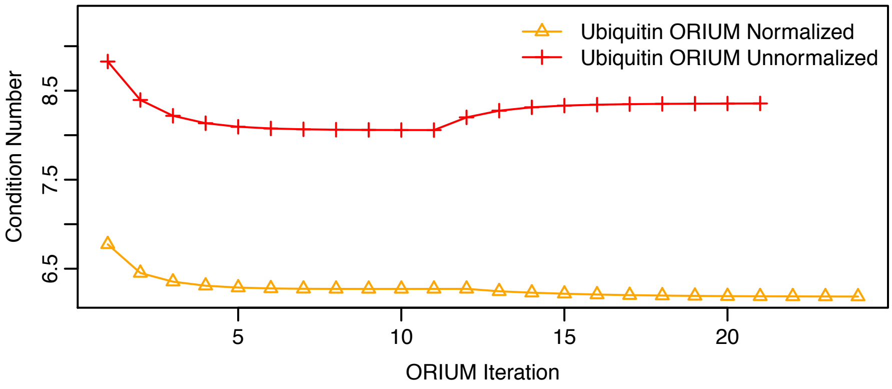

Figure S4: Comparison of condition numbers for ubiquitin determined from ORIUM implemented with equation 24 (red) versus equation 25 (orange).

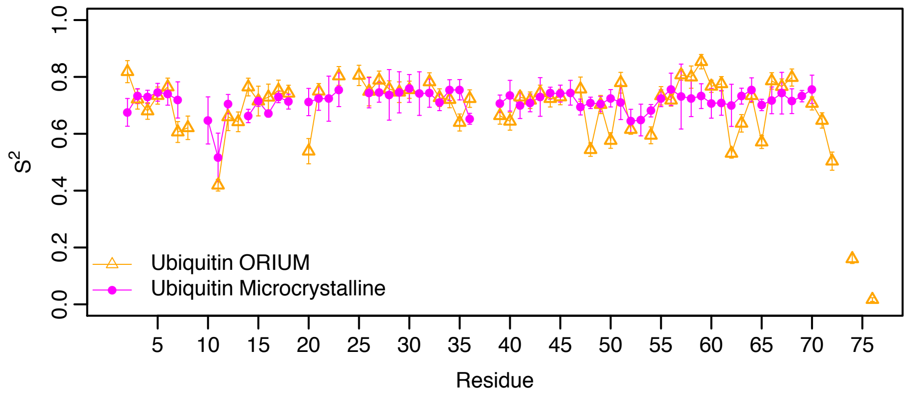

Figure S5: Comparison of N-H^N order parameters for ubiquitin from ORIUM (orange) and in the microcrystalline state (magenta) (Schanda et al. 2010).

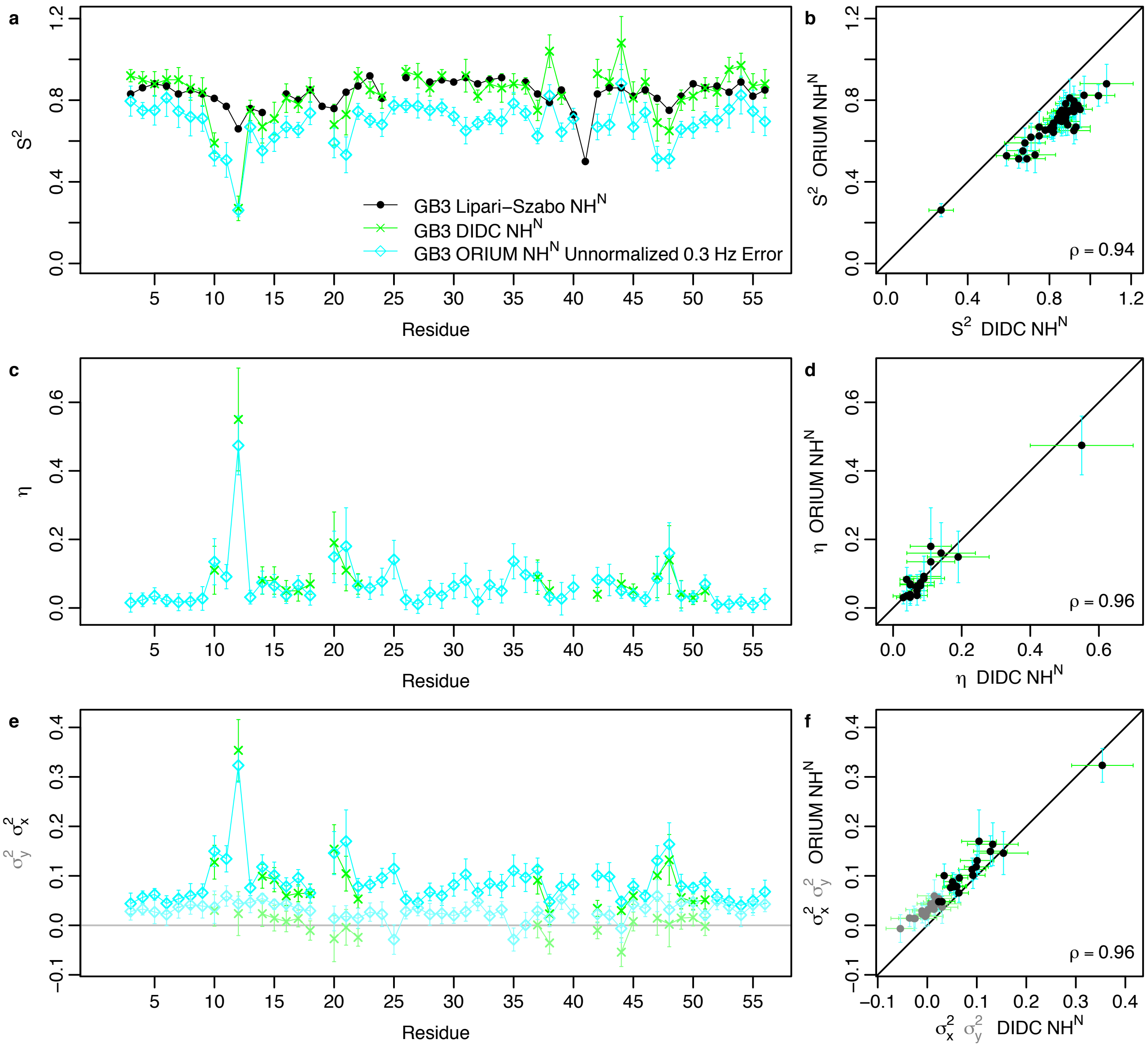

Figure S6: Comparison of ORIUM using equation (24) (cyan) and iterative DIDC (green) derived N-H[N] S^2_{RDC} and η parameters for GB3 (Yao et al. 2008). The ORIUM calculation was performed with both the N-H[N] and Cα-Hα RDCs simultaneously. For ORIUM (DIDC), the estimated error results from 1000 (100) Monte Carlo simulations that add uncertainty to the RDCs drawn from a Gaussian distribution with a standard deviation given by the error used in the iterative DIDC publication for the MC simulations (0.3 Hz). From the iterative DIDC analysis, η is determined only for residues that were not fit to an isotropic motional model. (a): S^2_{RDC} plot by residue. The solid line represents the S^2_{LS} parameters (Hall and Fushman 2003). (b): S^2_{RDC} correlation plot. (c): η plot by residue. (d): η

correlation plot. (e): σ_x^2 and σ_y^2 (lighter colors) by residue. (f): σ_x^2 and σ_y^2 (lighter colors) correlation plot.

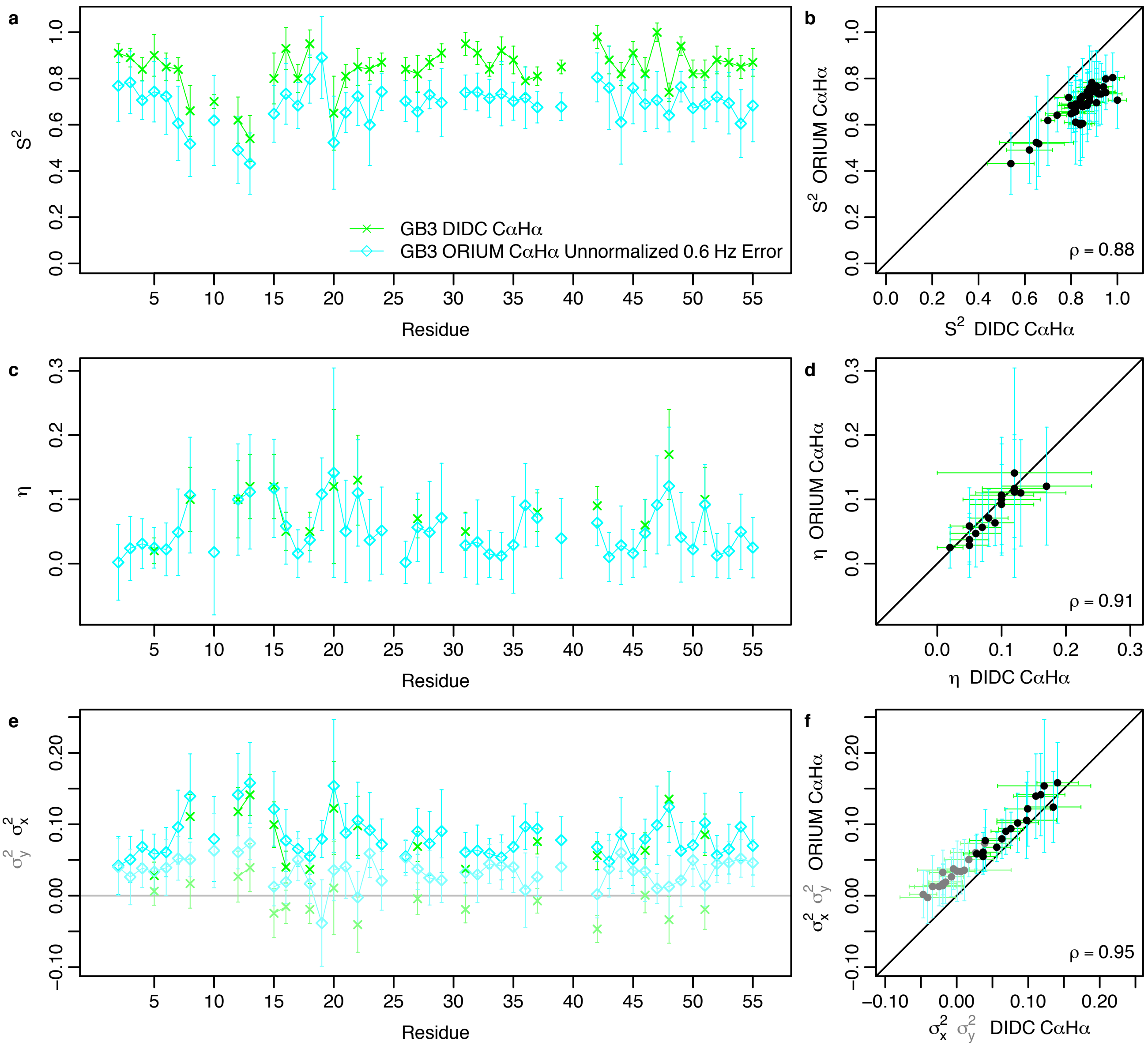

Figure S7: Comparison of ORIUM using equation (24) (cyan) and DIDC (green) derived Cα-Hα S^2_{RDC} and η parameters for GB3 (Yao et al. 2008). The ORIUM calculation was performed with both the N-H^N and Cα-Hα RDCs simultaneously. For ORIUM (DIDC), the estimated error results from 1000 (100) Monte Carlo simulations that add uncertainty to the RDCs drawn from a Gaussian distribution with a standard deviation given by the error used in the iterative DIDC publication for the MC simulations (scaled down to 0.3 Hz). From the iterative DIDC analysis, η is determined only for residues that were not fit to an isotropic motional model. (a): S^2_{RDC} plot by residue. (b): S^2_{RDC}

correlation plot. (c): η plot by residue. (d): η correlation plot. (e): σ_x^2 and σ_y^2 (lighter colors) by residue. (f): σ_x^2 and σ_y^2 (lighter colors) correlation plot.

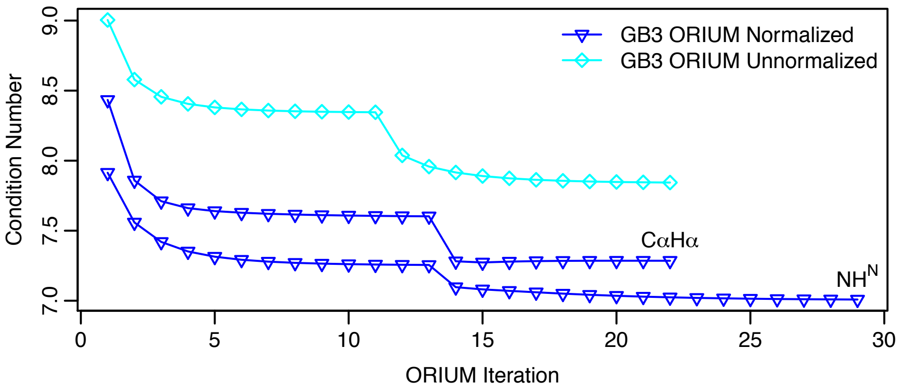

Figure S: Comparison of condition numbers for GB3 determined from ORIUM implemented with equation 24 (cyan) versus equation 25 (blue).
